# Is there any (emotional) difference between seeing art in the museum vs. in the lab? A comparative study of phenomenal art experiences of original artworks and digital reproductions

**DOI:** 10.3389/fpsyg.2026.1766700

**Published:** 2026-09-08

**Authors:** Stephanie L. Miller, Alexandra Victoria Alvarez, Rebekah Rodriguez-Boerwinkle, Matthew Pelowski

**Affiliations:** 1 Faculty of Psychology, University of Vienna, Vienna, Austria; 2 Department for Arts and Cultural Studies, University for Continuing Education, Krems, Austria; 3 Yale Center for Emotional Intelligence, Yale University, New Haven, CT, United States

**Keywords:** art viewing, digital reproductions, emotional experience, latent profile analysis, museum study

## Abstract

**Introduction:**

In the current post-digital age, technology is omnipresent in our everyday lives and digital encounters with (even originally analog) art are increasingly pervasive. Museums, artists, and art professionals continue to utilize digital technologies in their practices, and both access to and awareness of digital platforms continue to expand. In spite of this, little is known about how experiences with digital reproductions on a computer actually compare to original artworks in a museum, especially regarding viewers' global, felt-experiences. Previous investigations of digital art experiences report rather small and inconsistent differences between museum and laboratory conditions. These studies have tended to focus on hedonic responses and aesthetic judgements, and of these, few include comparative museum conditions with high-quality, genuine works of art.

**Methods:**

In the current study, we present a direct comparison of first-hand experiences of artworks in the gallery and in-lab experiences of their digital surrogates. We used a previous large-scale collection of museum experiences as a basis for this comparison, selecting eight artworks from the original study to digitize and present to new participants in a traditional laboratory setting. Initial results replicate previous findings, where aesthetic judgement ratings were found to be significantly, but only marginally, higher in the museum condition. In addition to collecting standard judgement ratings, we also employed a new self-report measure for capturing phenomenal experiences with visual art encounters that uses confirmatory latent profile analysis to identify and assign individual reports to one of five established experience types: Harmonious, Novel, Transformative, Disengaged, and Negative.

**Results:**

We found a small but significant difference in the frequency of experience types between the museum and digital cohorts, with the latter producing more Disengaged and Novel experiences, and lower rates of Harmonious and Negative experiences. A significant interaction between setting and experience type was also found for Negative and Transformative types.

**Discussion:**

Our study shows that artworks that cluster around negative affect experience types are better supported within the scaffolding of a museum setting.

## Introduction

Digitization has transformed the way that visual art is produced, circulated, consumed and—quite potentially—experienced. While once typically requiring a physical visit to a gallery or museum, art viewing can now increasingly be accomplished on the internet, through various social media, or via a wide variety of other digital interfaces and technologies. Museums are not just digitizing their collections but reimagining what an art experience can be through digital technology—making their vast catalogs available for download, sharing their artworks via multiple devices, and providing virtual tours (Romano, 2025). Artists, both established and emerging, have likewise increasingly turned to digital modalities (Cascone, 2023; Darcy, 2023; Mitchell, 2024). Today, 92% of museums offer virtual programming (Cuseum, 2021), and almost all museums have a website and social media presence, as well as some means of online viewing, and routinely utilize digital modalities on their premises (2024 National Snapshot of US Museums, 2024; Li et al., 2024).

This change from “analog” and in-person to digital and often remote delivery, in turn, reflects a number of intriguing changes and potentials throughout artistic, curatorial, and visitor spaces. While these developments have been accelerated by the recent COVID-19 pandemic (Cao, 2024; Li et al., 2024; Radermecker, 2021; Samaroudi et al., 2020; Trupp et al., 2022), the present state of digitalization has been building for many years (Hume and Mills, 2011). Museums, curators, artists, collections teams, and the art market have always worked alongside technological progress to innovate and offer novel experiences to global audiences (Karp, 2004). At the institutional level, digitization is suggested to serve multiple institutional goals, including increasing access for those limited by mobility, location, or economic barriers (Trupp et al., 2022). Digitization may aid preservation efforts by minimizing the handling of fragile works (Li et al., 2024) and enable educational outreach and research (Darda et al., 2025; Navarrete and Mackenzie Owen, 2016). It may further open up new avenues for curatorial experimentation (Cameron, 2007; Li et al., 2024), serve as a democratizing tool that strengthens transparency and public participation (Hylland, 2017; Karp, 2004), and support emerging initiatives—especially for individual-centered, targeted delivery—for addressing health, wellbeing, and other societal challenges (Bu et al., 2022; Clayton and Potter, 2017; Cotter et al., 2025; Trupp et al., 2022, 2023).

For viewers, the digital movement also represents key potentials–from a wider selection of art-viewing choices beyond the confines of economics or geography, to the ability to seek out new, individually-tailored, but also novel and surprising content. This may also provide a more intimate means of engaging the arts, fitting into the ways that many individuals increasingly consume or seek-out entertainment and culture (Bu et al., 2022; Cameron, 2007; Unitt, 2020). Digital presentations may also represent the way that we may first discover or even search for art; online thumbnails, PDF previews, Instagram posts, WhatsApp images are a common presence in most current societies. Digital proxies are even guiding buying decisions, as recent trend reports suggest that up to 43% of commercial galleries and 73% of art collectors first engage a digital image as a means of presenting or selecting artworks (Kakar, 2025).

At the same time, and despite this clear shift toward digitization, engagements with digital modalities are still, rather surprisingly, under-researched. This includes basic questions such as how (and whether) experiences of visual artworks may actually differ or be specifically impacted by digital vs. “real,” in-person contexts.

Empirical studies on the arts do, of course, have a longstanding tradition of laboratory studies, which typically involve digital or other (i.e., in early studies, printed) reproductions, as they allow for assessments in controlled, focused settings (Pelowski et al., 2017a,b) while still empowering—presumably—insights into how art is appreciated and experienced. However, only few studies have directly considered the role of context, or setting, in shaping art experience (Brieber et al., 2015a,b; Grüner et al., 2019; Specker et al., 2023a,b) or have directly compared the same art between digital/lab interactions to original artworks in the museum (Darda et al., 2025; Jonauskaite et al., 2024; Pelowski et al., 2017a; Specker et al., 2017; Szubielska et al., 2021). The limited body of existing research is made even more surprising, and pertinent, because, in addition to the potential *benefits* and use cases enabled by digital delivery, there are also longstanding arguments that digital reproductions should be, in some way, *sub-optimal* or *different* (Alelis et al., 2015; Cameron, 2007; Hume and Mills, 2011; Salas Espasa and Camacho, 2025). Since the turn of the 20th century, writers have argued that there must be especially something special about viewing real art in a museum context, with, for example, Walter Benjamin arguing that technological reproduction fractures the “aura” of an artwork, or its unique presence in time and space (Benjamin, 1936). Contemporary museum scholars and art-critics question how digital surrogates might alter perception and engagement, with both said surrogates and physical objects in the museum (Schweibenz, 2018) and art professionals tend to treat original artworks as ontologically and experientially distinct from reproductions (Cameron, 2007).

Recent psychological discussions align with this view and suggest that art might need to be seen in person for its full effect to be experienced, and that digital formats or other reproductions “lose” necessary qualities—visual appeal, interestingness, immediacy, ambiance, level of engagement, and perceived importance—when viewing original vs. reproduced art examples (e.g., Benjamin, 1936; see also Berger, 2008; Pelowski et al., 2017a; Specker et al., 2017; Trupp et al., 2023). These arguments are paralleled also by widespread recognition that individuals *do* often make special trips to museums, our societies set aside significant resources for art display and collection, and we often feel that the opportunity to see the “real things” in their native conditions must be taken when visiting a new city or country (Brieber et al., 2014, 2015a,b; Duncan, 1995; Falk and Dierking, 2016; Locher et al., 1999, 2001; Locher and Dolese, 2004; Pelowski, et al., 2017a). Experiencing art in a museum typically involves the conscious decision to travel to such a setting, pay an entry fee, all while being surrounded by other visitors typically within a rather opulent building or “white-cube,” designed institution (Cameron, 2007; O'Doherty, 1986). Such tangible aspects of an in-person visit as well as, even more basically, taking a break from everyday life or even the physical exertion of walking around a museum (Carbon, 2017; Chatterjee and Noble, 2017) have been proposed to drive some of the positive impacts museum-goers experience. If true, these might be lost or diminished in digital presentations.

Among the handful of empirical studies that have been conducted, several have indeed suggested that digital formats, when compared to in-person gallery viewing, may lead to some generally lower ratings of, for example, pleasantness (Locher et al., 1999, 2001; Locher and Dolese, 2004), interest (Locher et al., 2001; Locher and Dolese, 2004), liking, time spent viewing, positive emotion, or arousal (Brieber et al., 2014, 2015b). However, these studies, which to date have typically considered only aspects such as artwork ratings, preference, or understanding, also tend to report few actual significant differences. When differences do emerge between original and digital contexts, they are generally accompanied by negligible to small, and even inconsistent, sizes of effect. For example, Darda et al. (2025) found no significant differences in liking or beauty ratings of artworks or artifacts viewed in museum vs. laboratory settings. Work from Estrada-Gonzalez et al. (2020) found few contextual effects on eye tracking and fixation behaviors or aesthetic evaluations, and when differences did emerge, they were moderated by artwork properties rather than context itself. Furthermore, another study that attempted to disassociate the role of physical context and genuineness did not find significant differences in evaluations of artworks between a gallery and laboratory setting (Brieber et al., 2015a). A recent meta-analysis by Specker et al. (2023b) of the past comparative studies suggested in fact little evidence for a systematic effect of genuineness (i.e., viewing an original artwork vs. a reproduction) and inconsistent moderating effects of context (i.e., experiencing art in a museum vs. laboratory setting).

This leaves the study of art and the effects of digitalization in a rather interesting impasse. As the art world is increasingly pivoting to digital-first models, how do we understand what happens in the viewer's mind and body during these encounters? What are the potential affordances of using digital media in research? Do these actually not exist?

### Beyond basic hedonic evaluations or interest—Are there better, currently unasked candidates for digital vs. museum differences?

Although past studies have been inconsistent in finding actual differences when engaging digital formats, this could also be a product of how we have been making assessments and the kinds of questions being asked. Among other possible interpretations of the inconsistent effects found in their meta-review, as well as others that address questions of context and/or genuineness (Specker et al., 2023b) note that these studies tend to focus on hedonic assessments (e.g., valence, arousal, pleasantness) or meaning and understanding. While these are the most assessed responses in empirical studies of the arts (Brieber et al., 2015a; Forster et al., 2013; Mikuni et al., 2024; Nadal et al., 2010; Newman and Bloom, 2012; Sidhu et al., 2018; Trupp et al., 2023), previous research suggests that individuals are often quite good at looking past presentation format when making such evaluative judgements. For example, the facsimile accommodation hypothesis (Locher et al., 2001; Specker and Leder, 2022) proposes that while viewers recognize that a reproduction is not an original artwork, they mentally compensate for the reproduction's limitations when providing ratings. Viewers might look past differences in size, material, texture, and color because the reproduction is evaluated based on how they might imagine the original to be, rather than on the reproduction itself.

In their discussions of why it may not be surprising to find high consistency in these ratings across conditions, Specker et al. (2023) go further to suggest that past studies may simply have been focusing on the wrong candidates for detecting a difference. Rather than basic ratings or understanding of art, a better target may be found in the global, nuanced, and especially emotional experience. For example, Locher et al. (1999, 2001) compared slide-projected images of paintings (later followed up with postcard-printed images; Locher and Dolese, 2004) against the corresponding originals at the Metropolitan Museum of Art. They found that although evaluations related to pictorial content or composition were generally similar across presentation formats, viewers, irrespective of art background or training, evaluated original artworks as more surprising, rare, and immediate. Specker et al. (2017) directly compared people's aesthetic experience of the same artwork in the laboratory (reproduction) and the museum (using the Unusual Aesthetic Experience Scale, which assesses intense reactions such as awe or crying; Silvia and Nusbaum, 2011) and found that the art experience was generally more intense in the museum as compared to the laboratory.

Such findings are supported by theoretical discussions (Jacobsen, 2006; Pelowski, 2015) also suggesting that nuanced emotional reactions may be a key aspect of real, in person art. Scholars have further argued that current approaches to studying the art experience cannot hope to capture aesthetic responses arising from the interplay of emotion, cognition, expectations, physical movements, and longer, more open-ended interactions, which may lead to quite different outcomes (Augustin et al., 2012; Brown and Dissanayake, 2018; Cross and Ticini, 2012; Tallis, 2008). However, systematic comparisons at the emotion and especially the more nuanced, global experience level have not yet been empirically conducted.

Arguments can also be made for the types of art, and institutions, investigated. Many past studies using comparisons have also employed lesser-known art from smaller galleries or museums, or certain media (e.g., photographs; Locher and Dolese, 2004) that may not be candidates to really show a difference (see e.g., Pelowski et al., 2017a for review), as such works may not generate the strong expectations and cultural associations surrounding authenticity for significant differences to emerge (Newman and Bloom, 2012). Rather, when thinking of the types of museums and examples of art that many individuals do think of, and report as special, these are often more notable, grand, masterpieces of art (Duncan and Wallach, 2019).

All of this said, it may be that we do not in fact find a difference, even when considering more emotional, visceral, embodied aspects with art. Some previous study, on for example renderings of artifacts (Alelis et al., 2015) have suggested that regardless of age, digital modalities encourage emotional responses, and that engaging with digital reproductions did not lessen enjoyment or emotions felt. However again, research at large has not been able to significantly characterize shifts in mediality on visitor engagement.

### Present study

This study was designed to offer a more expansive comparison of art experiences with original artworks in the museum and digital reproductions in a laboratory setting. Previous findings suggest that we might better locate what is being lost, gained, transformed, or held constant when an artwork goes through digital reproduction if we center our inquiry on viewers' emotional, affective, and cognitive experience, rather than focusing solely on the material conditions of the artwork, hedonic assessments, and evaluative measures (Specker et al., 2023b). As such, our approach here focuses on individuals' nuanced, felt emotional, and phenomenal experiences, by employing a new method and utilizing previous data collection for systematic comparisons. Specifically, our approach sought to aggregate phenomenal/emotional state ratings into a single categorical variable of “Experience Type,” derived through confirmatory latent profile analysis. This variable can then be used in further analyses to efficiently capture complex patterns of self-reported emotional experiences and compare them across conditions.

Emotions, and felt affective and embodied responses in general, have been long connected to experiences of the arts. Across philosophical aesthetics and empirical psychology, from early theories of visceral empathy or “Einfulung” (Vischer, 1994) contemporary models of affective psychology (Pelowski et al., 2017c; Vartanian and Chatterjee, 2022) and empirical studies (Darda et al., 2025; Estrada Gonzalez, 2023; Fingerhut and Prinz, 2020; Menninghaus et al., 2019; Pelowski et al., 2018; Rodriguez et al., 2024; Specker et al., 2021), it has been well established that artworks can elicit a wide range of emotional responses in viewers. Reported experiences can vary widely, from detached and disinterested, intensely positive or severely negative, to challenging and transformational (Chrisafis, 2011; Csikszentmihalyi, 1991; Funch, 1997; Pelowski, 2015; Pelowski and Akiba, 2011; Sherman and Morrissey, 2017; Silvia, 2009; Smith et al., 2017). Various emotional responses can also be further linked to higher-order impacts of arts engagement, including effects on wellbeing and perspective change (Cotter et al., 2022; Igdalova et al., 2025; Pelowski et al., 2024, 2025; Rodriguez et al., 2024; Trupp et al., 2023). Following, for example, expressive theories (Ekman et al., 1980; Keltner, 2015), reported emotions and phenomenal states may provide a salient “roadmap” or indication of underlying psychological, cognitive, or even neurophysiological processes underlying interactions (Miller et al., 2024; Pelowski et al., 2017c).

Emotional and other felt-responses are also suggested to be a critical feature of art experiences that are susceptible to change when the setting or context shifts (Pelowski et al., 2017b), a point echoed and raised by the earlier review by Specker et al. (2023b; see also Darda et al., 2025) which proposed that emotions may be key candidates for identifying possible differences. Emotions in this application are not only psychological states linked to specific physiological responses and cognitive appraisals, but also embodied phenomena that are historically shaped, culturally mediated, and socially shared (Benjamin, 1999; Leder et al., 2004), thus offering a rich and accessible lens through which we might seek to understand art encounters across diverse contexts, viewing conditions, and (digital) medialities (Wetherell, 2012). Beyond classic emotions, aspects involving the body, body awareness, sense of immersion and presence, are among those typically suggested for what may make a “real” engagement with art in the museum special and different than when the analog artwork itself is duplicated in a digital or other mediality (Brieber et al., 2014; O'Doherty, 1986; Pelowski et al., 2017a). Assessing not only individual emotional states, but possible patterns in affective responses may further provide a more holistic means for considering potential differences in art experiences at the individual engagement level and in different conditions. Thus, this paper considered a robust range of emotional responses to both original artworks in their museum installations as well as to digital surrogates in a computer screen viewing modality.

To assess the nuanced phenomenal experience of these artworks, we employed a new measure and method that builds directly on a previous, large-scale data collection of individual art experiences with a range of visual art and within museums across North America and Europe (see Miller et al., 2025). This prior project asked everyday museum visitors to engage with a specific target artwork, then immediately report on what they felt while viewing that artwork, according to a measure that included a range of both previously art-related and more general emotions, as well as epistemic, meaning-related, and bodily responses. Collected reports were combined via network modeling to map the reported experiences and identify associations between groups of and individual items. A set of 16 “core” items was then selected with the aim of summarizing the global network structure. Responses to these items were input to a latent profile analysis (LPA) that examined potentially shared, underlying patterns in participant reports and subsequently identified five response profiles that reflected (1) a *Harmonious* experience characterized by above average ratings of positive affect terms, (2) a *Novel* experience in which high positive affect was accompanied by reported self-reflection, (3) a *Transformative* outcome in which self-reflection was instead paired with high ratings of negative affect terms, (4) a *Disengaged* experience distinguished by low ratings of all items, with the exception of “boredom,” and (5) a *Negative* outcome in which participants reported high levels of only negative affect terms. Beyond identifying the specific profiles, which largely matched foundational theoretical models (VIMAP; Pelowski et al., 2017c), each participant was further assigned to a profile group according to the coherence of that profile with their individual response pattern. Importantly, the character of each profile was stable across different artwork-viewer combinations, but their prevalence, or frequency, was found to meaningfully differ between artworks (Miller et al., 2025).

The five identified profiles, or experience types, can be thought of as representing specific, supraordinate varieties of phenomenal art experience and offer a strong understanding of nuanced, but also shared, responses to art. Collectively, they describe the possible experiential “endpoints” different individuals may reach with one artwork, through an overall flow of processes that is theoretically connected by a common, shared basis at the psychological level across specific meetings of individuals, contexts, and artworks. This theoretically-guided framework offers a novel approach for assessing individual art encounters that can consider a high level of nuance and mix of processes and factors (Miller et al., 2024; Pelowski et al., 2025). The project further provided several tools that can be applied to future investigations, including the refined list of 16 “core” items, which can serve as a means of quickly identifying experience types with new participants and artworks, and a lexicon of well-tested artworks with documentation of expected distributions of experiences type (Miller et al., 2025).

We now directly compare reported phenomenal experiences of original artworks in a museum to those of digital reproductions on a screen in a traditional laboratory setting. Building on the original project from Miller et al. (2025), and integrating our present focus on experiences across settings, we conducted a comparative laboratory study with a subset of eight artworks chosen from the original set. Participants engaged with digital reproductions of the selected artworks using available laboratory desktop computers and reported on their experiences using the identified list of 16 “core” items from Miller et al. (2025), as well as common aesthetic appraisals and impact measures (e.g., liking, meaningfulness, hedonic wellbeing). By selecting from same artworks and response questions, we were able to directly compare the reported experiences of those who viewed the genuine artworks in the museum to those who viewed the artworks digitally in the laboratory. We first assessed baseline differences in reports between the Museum and Digital groups, in an effort to identify general effects of setting in a similar manner as previous studies. We then conducted a confirmatory latent profile analysis (CLPA) with the newly collected phenomenal reports from the Digital group to assign participants to the previously established experience type profile groups. In applying this method to our current inquiry of potential differences between original-museum and digital-lab art experiences, we can not only compare the frequency at which these profiles occur between artworks and settings but consider variation in the effects of setting on aesthetic judgements and other ratings across experience type groups.

## Materials and methods

The present study involved two separate data collections, connected at the level of engagements with the same works of art. These included: (1) our earlier collection of individuals' interactions with a selection of museum art. This collection (hereafter “Museum” group; see also Miller et al., 2025 for full description of methods) was conducted as part of our above-mentioned program in which we assessed a large, representative dataset of museum-based art engagements by targeting participants' reported felt experiences and identified shared phenomenal patterns or art experience outcome types. For the present investigation, we revisited a subsample of the Museum dataset, targeting engagements with eight of the original 31 artworks. These specific pieces were chosen to represent a good mix of art styles and identified experience response types, and the original artworks—and accompanying individual viewer reports, appraisals, and other interpersonal information—served as a basis for a new comparison to the digital surrogates.

(2) Digital reproductions of the eight selected artworks were sourced online, exhibited in a lab-based computer modality, and presented to a new cohort of participants (hereafter “Digital” group; see Table 1 for detailed information on the selected artworks). We employed the same general procedures and emotion/phenomenal state questions but with all artworks shown in a between-participants design and in order to compare responses at both the individual emotion and also broader experience type level. This approach allowed us to target the question of how especially phenomenal factors might be differentially impacted by the original, museum and digital, laboratory conditions.

**Table 1:** Descriptive information of stimulus artworks.

Title	Artist	Year	Institution (Country)	Style	Museum cohort *N*
La Hara	Jean-Michel Basquiat	1981	Albertina (AT)	Neo expressionism	87
Orange Hat II	Alex Katz	1973	Albertina (AT)	Pop-realism	100
Rising Forms	František Kupka	1922–23	Albertina (AT)	Abstract	90
The Enchanted Domain	Rene Magritte	1953	Albertina (AT)	Surrealism	111
Birds and Insects	Joan Miro	1938	Albertina (AT)	Abstract/Surrealism	100
The Water Lily Pond	Claude Monet	1917–19	Albertina (AT)	Impressionism	97
Number 19	Mark Rothko	1949	Art Institute of Chicago (US)	Abstract expressionism	79
Christ on the Mount of Olives	Paul Troger	around 1750	Belvedere (AT)	Baroque	81

Information on all artworks included in the reported studies (*N* = 8). All were examples of two-dimensional paintings. Throughout the main text, and further tables and figures, artworks are referred to by the artists' last name. Listed by-artwork samples sizes of the Museum cohort (M = 93.1 per artwork) are following participant exclusion for incompleteness or inattention. The Digital cohort included N = 113 participants for each artwork; no participants were excluded. AT, Austria; US, United States.

### Participants

The overall combined study involved *N* = 858 participants between the two cohorts. The “Museum” cohort included a final sample of *n* = 745 individuals (57.7% female; *M*_age_ = 34.64, *SD* = 15.30; see Table 2 in Results). This group, recruited as part of the earlier Miller et al. (2025) paper, involved all of the participants who had engaged with one of the eight artworks selected for the present study (the full sample for the previous project involved *N* = 2,747 individuals across 31 artworks). Each participant in the Museum condition viewed and reported on their experience of only one artwork, with an average of 93.12 unique viewers for each of the eight selected artworks (*SD* = 10.84, range = 79–111). The decision to use a between-participants design for this earlier data collection was based on the aims of the original study. This also provided quality artworks and rich data for use in the present study when considered at the individual viewer experience level.

**Table 2:** Comparison of condition group demographics.

Demographic	Condition				χ^2^-statistic (*df*)	Significance (*p*)
	Museum		Digital			
Gender					13.241 (2)	0.0013^**^
% female	57.7		72.6			
% male	40.8		23.9			
% non-binary/other	1.5		3.5			
	***M***	***SD***	***M***	***SD***	***t*** **-statistic (** ***df*** **)**	**Significance (** ***p*** **)**
Age	34.6	15.30	28.3	8.22	−6.6392 (251.13)	< 0.001^***^
General liking of art	8.08	1.69	7.72	1.97	−1.8771 (138.84)	0.0626
General knowledge of art	5.00	2.10	4.79	2.24	−0.93347 (143.72)	0.3521
Museum/gallery visits per year	7.24	15.2	5.63	6.44	−1.9532 (348.19)	0.0516

Museum descriptives reflect the full cohort, including respondents to each of the eight artworks: total *N* = 745 (M_artwork_ = 93.12, SD = 10.84, range = 79–111). Digital condition descriptives reflect *N* = 113 participants, each of whom viewed and responded to all eight artworks. *p < 0.05. **p < 0.01. ***p < 0.001.

The number of unique viewing instances per artwork example (i.e., roughly *n* = 100) followed best practice suggestions for the analyses utilized in the original study (Christensen and Golino, 2021; Golino and Epskamp, 2017). All participants in the Museum group were convenience-sampled from the everyday visitors of the target museums (see Table 1). The sample population included a wide range of individuals, the demographics of whom generally aligned with typical sociodemographic profiles of museum visitors in European and North American contexts (Falk and Katz-Gerro, 2016; Pelowski et al., 2017a; Tinio et al., 2014). Individuals were not compensated for their participation.

For the Digital comparison, we recruited a new sample of *n* = 113 participants (72.6% female; *M*_age_= 28.3, *SD* = 8.22). These individuals were similarly recruited from a general (i.e., non-student) population and with the aim of generally matching the broad demographics of the museum visitor populations. Recruitment was done online, via a Study Participant Platform of the University of Vienna (built on the hroot software; Bock et al., 2014), which is composed of individuals from the greater Vienna city population who had shown interest in participating in social scientific studies for monetary compensation. Notably, as the majority of our selected artworks were originally displayed in museums of the same city, this further allowed for a good between-cohort comparison.

The sample size of the Digital cohort was based on the same suggestions for unique viewing instances, at the stimuli level (i.e., *n* = 100, Golino and Epskamp, 2017; Christensen and Golino, 2021) and taking into account the planned within-participants design, where all participants would have an opportunity to view all eight works. Thus, the (smaller) sample both provided a general equivalence to the previous Museum study, while addressing pragmatic needs for reasonable sample size logistics. We also included a budgeted over-sampling of 20% to account for possible dropouts or unusable data; ultimately no participants were excluded. Individuals were compensated €10 for their participation.

Both the original and present studies followed all protocols of the ethics board of the University of Vienna and adhered to standards set by the Declaration of Helsinki.

### Stimuli

Stimuli for the present study involved eight works of art from highly accomplished and esteemed artists selected from the earlier collection of 31 museums artwork on view in leading museums in Europe and North America (see Table 1 for details on the eight selected artworks). Despite the museum variety, all artworks were displayed in standard presentation formats, centered at eye level typically 145–152 cm from the ground, on solid color backgrounds. The artworks were specifically chosen due to their medium type (two-dimensional paintings), broad range of periods and styles (including Baroque, Surrealism, Abstract, and more), and expected variance in phenomenal experience-type profiles—based on the original Museum cohort analysis (see Results below).

The digital reproductions were all downloaded at their highest available resolution, sourced from the museum's public online collections or through partnered affiliations with open access to content, color-checked, and processed in Adobe Suites to preserve the original aspect ratio. They were resized and proportioned to fit the Qualtrics presentation frame (1800–2100 px) width for a 16:9 monitor, ensuring large, non-scrolling, undistorted presentations for uniform display across trials, and displayed on a black background. This preparation allowed for the Digital cohort to engage with each artwork in a computer screen exhibition style format that maintained composition structure, relative scale, and visual detail, consistent with a museum format.

### Procedure

The overall procedure for the study used a mixed design, with the main target of “condition” (original artwork in the museum vs. digitized in the lab) compared between our participant groups; “artwork” was compared within participants in the Digital cohort (as all participants viewed all artworks) and between participants in earlier Museum cohort (as they had each seen only one of the artworks).

#### Museum cohort

For the original Museum group, we had employed a procedure based on best practices in empirical museum studies and which aimed to best isolate a single artwork for a viewing experience (see Miller et al., 2024 for review of procedural practices). At each museum site, visitors were first approached by a member of the research team outside the entrance to a gallery holding one of our preselected artworks. This contact was aimed at meeting potential participants early in their museum visit or before entering a new section of the museum in order to minimize previous exposures or fatigue (Bitgood, 2009). Visitors were asked if they would be interested in participating in a study about their appreciation and experiences with a specific work of art. If they agreed, participants were directed to a pre-selected, target artwork and instructed to view this however they usually would while visiting a museum and for however long they liked. They were further instructed to immediately return to the researcher when they had finished viewing the artwork, at which time they completed a post-viewing questionnaire outside the gallery space (including demographics, phenomenal art experience, and other ratings; see Measures below). Participants could complete the survey on paper or on their smartphones via a QR code and the online survey platform Qualtrics (Qualtrics, n.d.). Survey questions could be completed via either format in English or German. Due to procedural constraints in the museum venues, artwork viewing time were not be specifically tracked.

Importantly, again, participants in the Museum group viewed only one artwork, were left unobserved during engagement, and completed all survey questions after viewing. Participant selection was further constrained to only single or paired adults, omitting larger (e.g., tour) groups and individuals accompanying children, as these cases have been shown to lead to differing (i.e., social or educational) varieties of engagement (see Goulding, 2000; Chang, 2006; Pelowski et al., 2017a). This procedure has been previously shown in several studies to provide a good basis for assessing art experiences in an ecologically-valid, albeit reasonably controlled, environment, and especially for assessing reported emotional, or other felt, experiences (Kühnapfel et al., 2023; Miller et al., 2024; Pelowski et al., 2024). More detailed information on the procedure followed by the Museum group can be found in Miller et al. (2025).

#### Digital cohort

For the Digital viewing condition, we aimed to duplicate the same basic viewing and rating procedures as above, however following a typical lab-based study design (see e.g., Forster et al., 2013; Brieber et al., 2014; Mikuni et al., 2024). Upon arrival to the lab, participants were seated at a desk in front of a 2” monitor (LG 24MB65PM, 1920x1200). They signed an informed consent and completed a brief pre-questionnaire (including demographics, art interest, and a mood assessment).

Participants were asked to view one work of art at a time, presented as digital reproductions of the museum originals (see Stimuli above). All artworks were shown as standalone images, centered on the screen with a black background, and with no other accompanying information. Participants were instructed to view the work of art for as long as they wished and then to click an arrow button when they were finished. After the single artwork viewing, they were asked to report on their experience of and rate the artwork, with a brief survey largely matched to, but slightly adapted from, that completed by the Museum group (e.g., a reduced measure of emotional-phenomenal experience; see Measures below). This procedure was repeated until participants viewed and rated all eight artworks; the presentation order was randomized between participants. After viewing all 8 artworks, the viewing experience ended, and participants were asked to complete a post-viewing survey assessing their overall experience of the study and if/how they perceived their art experiences in the study to be similar or different to those that they had previously had in museums.

Artwork presentation and reporting was done via Qualtrics (Qualtrics, n.d.). Survey questions could again be completed in English or German. Participants in the Digital cohort spent on average 24.52 s looking at any particular artwork during the study (*SD* = 23.39, range = 1.21 to 213.30). This was generally in line with both museum and lab studies using the same free-viewing paradigm (Brieber et al., 2014; Smith et al., 2017; Tinio et al., 2014), including our past pilot study on encounters with individual artworks using the same phenomenal profile approaches and from the same target museums (Miller et al., 2024).

### Measures

The survey questions for both studies were as follows (see also Miller et al., 2025 for complete reporting of all survey items included in the original study).

#### Demographics

Demographic questions for both cohorts included age, gender, and nationality. Participants also responded to two questions regarding their general art interest and knowledge (“How much do you like art?,” “How knowledgeable are you about art?”; rated on Likert-type scales, from 0 = “Not at all” to 10 = “Very much”; Miller et al., 2024), as well as approximately how many times per year they visit museums or galleries (Miller et al., 2024; Pelowski et al., 2024).

Participants in the Digital group only also completed a brief mood assessment, including ratings of positive mood, negative mood, and general emotional arousal (rated on Likert-type scales, from 1 = “Low” to 7 = “High”; Mackinnon et al., 1999; Pelowski et al., 2024). Note again, all demographic questions were completed by the Digital cohort before the main study (i.e., art viewings); the Museum cohort completed demographic questions after viewing the target artwork, but before responding to any other questions about their experience.

#### Post-viewing phenomenal experience

The main behavioral measure for the study involved presenting participants with the prompt, “While viewing the artwork, I felt…” and 16 phenomenal terms, answered via 11-point unipolar scales (0, 1–10: 0 = “Not at all”; answers from 1–10 corresponded to some incidence of a feeling, as well as relative magnitude, with 10 = “Very much so”). The items (see also Miller et al., 2024, 2025 for further discussion) included a range of positive affect states (*love, happiness, playfulness, pleasure, beauty*), as well as states associated with (meta)cognition and reflection (*insight, being moved, a transformation, a change in myself, being thrilled, a sense of active engagement*), and negative affective states (*boredom, shock, anger, sadness, anxiety*).

**Figure 1:**
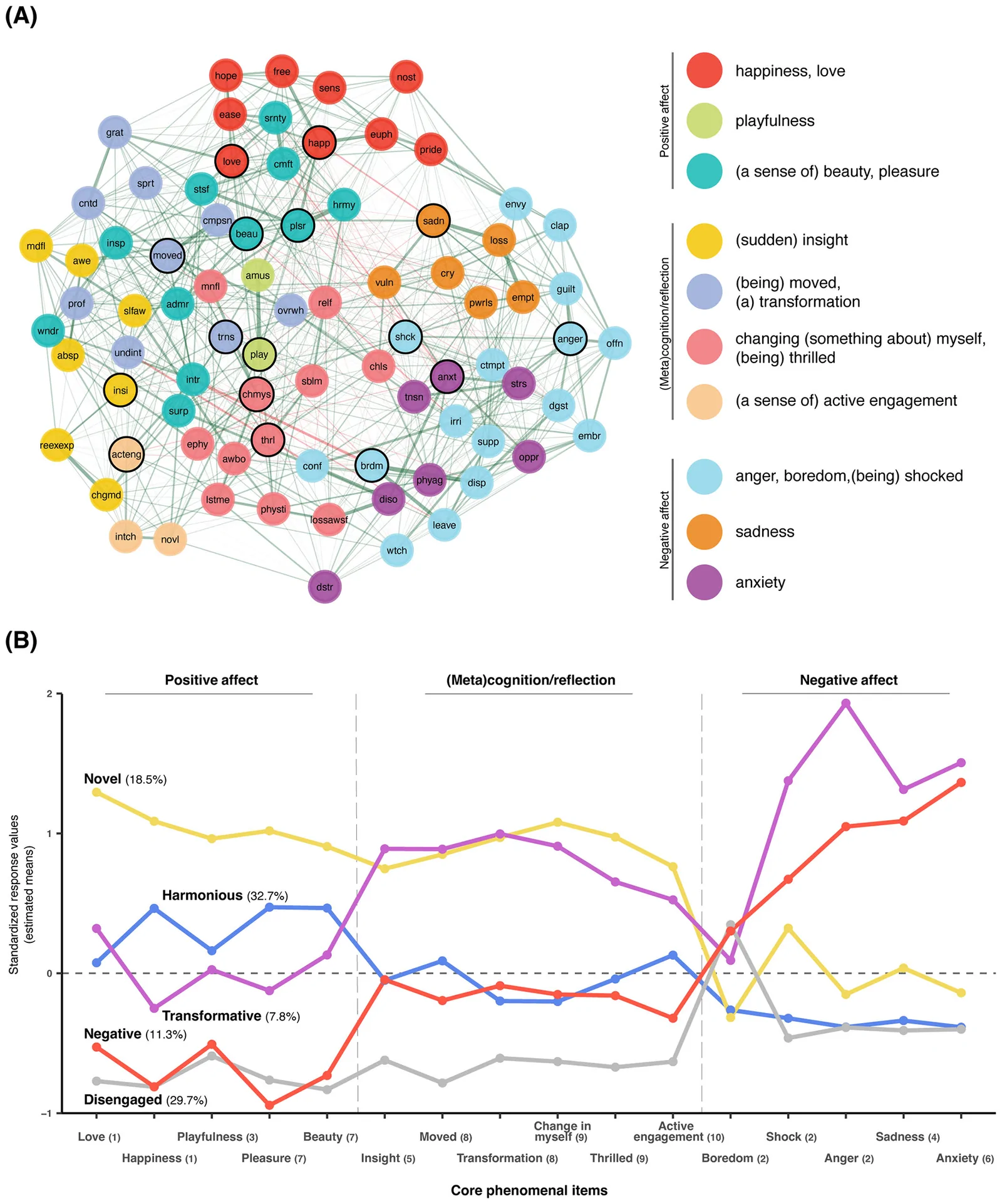
Network model and latent profile analysis results from prior large-scale collection of museum art experiences. Original data collection, analysis, and figures are published in Miller et al. (2025) CC-By Attribution 4.0 International. OSF Preprints (https://doi.org/10.31219/osf.io/ectbh_v2). Figures presented here are adapted for the current publication. **(A)** Prior network model, constructed via hierarchical exploratory graph analysis, from *N* = 2,747 reports of felt-experience across *N* = 31 artworks. Network includes *n* = 81 phenomenal terms, depicted by individual circles (i.e., “nodes”). Node color represents community belonging; connecting lines indicate Spearman rank-order correlations (green = positive, red = negative). Nodes with bolded outlines were identified as “core” items, according to hybrid centrality value rankings within each community, and were input to the subsequent latent profile analysis. **(B)** Prior results of a five-profile solution latent profile analysis. Points represent groupwise estimated mean ratings (standardized by-item to a sample-level *M* = 0, *SD* = 1); lines are included to aid legibility. Lower axis labels list the 16 core items identified from the network model. See Figures 4 and 5 of Miller et al. (2025) for full figure descriptions.

This set of items was again derived from Miller et al. (2025) and represented a reduced, core summary of the original list of 90 phenomenal states utilized in the large-scale collection of museum art experiences. Participant responses to these 90 items were combined into a network model, which described the phenomenal space of the collected art experiences (see Figure 1). The 16 “core” items were selected based on network metrics, and, as a reduced list which well summarized the global network structure, were input into a subsequent latent profile analysis (LPA), which identified underlying patterns to participants' responses, revealing five latent profiles (Miller et al., 2025).

Applying the list of 16 core items as a behavioral measure with the Digital cohort was expected to allow us to calculate and identify each participant's report of a given artwork as a specific ExpType, thus serving as a basis for comparison. See Results below for details on applying the previous LPA model to new experiential reports collected from the Digital group. This measure was administered immediately following each art-viewing experience that participants completed (either once in the Museum group after their single art-viewing, or after each individual artwork in the Digital group). While participants in the Museum group completed ratings of all 90 items, only responses to the 16 core items were considered in the present study.

#### Artwork ratings and impact measures

In addition to the above phenomenal ratings, participants were also asked to rate the artwork (“I thought the art was…”) according to a series of 7-point bipolar, semantic differential scales (Osgood et al., 1957), assessing *liking, beauty, goodness, meaningfulness, complexity*, and *interest* (Miller et al., 2024; Pelowski, 2015; Pelowski et al., 2018, 2024). Participants were then asked how they would describe their “overall experience of the artwork,” (7-point scale, 1 = “very visceral and emotional” to 7 = “more detached and cognitive”) and whether they would “pay to see the artwork again/in person” (7-point scale, 1 = “Definitely not” to 7 = “Absolutely, I would”'; Miller et al., 2024).^1^

Additionally, participants responded to three items (11-point scale, rated from 0 to 10) that asked to what degree their experience made them feel worse or better, made them a worse or better person, and if it changed them in any way (Miller et al., 2024). These were included in both studies as general indicators of possible (i.e., wellbeing related) impacts of the experiences and in order to provide some indication for broader importance of arts engagements.

The survey completed by Digital group incorporated one additional item in this section, in which participants were asked to rate for each artwork the extent to which they felt “immersed in [their] experience” (7-point scale, 1 = “Not at all immersed” to 7 = “Completely immersed”). This item was added based on previous studies that identify immersion as a dimension of digital arts engagement relevant to broader wellbeing and flourishing impacts of an experience (Alpys et al., 2025; Cotter et al., 2025).

#### Additional post-study questions on overall experience (Digital cohort)

Finally, participants in the Digital group also responded to several items following all eight art-viewing and rating tasks and referring to the overall experience and their relation to digital presentations. Participants first repeated the three-item mood assessment for matching to the pre-study answers. They were then instructed to think about their overall experience during the study and asked to rate the extent to which their “experiences today [were] similar to those [they] have had in museums” [7-point scale, 1 = “Not at all the same (completely different)” to 7 = “Not at all different (completely the same)”]. This was included as a general check regarding how they had viewed the engagement and whether this might generally coincide or differ from typical museum engagements (Duncan, 1995; Duncan and Wallach, 2019; Karp, 2004; Kemp, 1998; Malarux, 1974). They also rated how “visually pleasing,” “emotional,” and “immersive” their experiences during the study were compared to past experiences in museums [7-point scale, 1 = “Significantly less so (than museums)” to 4 = “Exactly the same” to 7 = “Significantly more so”].

Participants also rated the extent to which they believed specific features of a museum contributed to any differences they might have felt between their experience in the study and previous museum experiences (7-point scales, 1 = “Not at all” to 7 = “Very much”; Pelowski et al., 2017a). Features included “the atmosphere of the museum,” “the architecture of the museum,” “the social environment,” “a sense of immersion,” “being able to move freely,” “the size of the artwork(s),” “the visual quality of the artwork(s),” and “being able to see the real artwork.”

## Results

Each participant in the newly collected Digital group viewed all eight stimulus artworks, with no participant excluded at either the individual or trial (i.e., individual artwork) level, with 113 unique responses per artwork (see again Table 1 for by-artwork sample sizes from the Museum cohort). A comparison of the Museum and Digital cohorts across the full populations is provided in Table 2. Compared to the Digital group, the Museum group was on average slightly older and represented a more balanced gender distribution. However, notably, the cohorts reported comparable levels of general liking and knowledge of art. The groups similarly did not differ in frequency of museum visits and, once again, both groups generally aligned regarding typical sociodemographic indicators of European museum visitor populations.

### Possible order effects/mood—Digital cohort

At the start of the study session, participants in the Digital condition reported, on average, moderate to high positive mood (M = 5.31, SD = 1.06), low negative mood (M = 2.16, SD = 1.23), and moderate general emotional arousal (M = 4.43, SD = 1.26) and with no outliers or other suggestions of possible conflating factors. Pairwise *t*-tests showed that there was a significant, albeit minimal, decrease in reported positive mood, from the start to end of the study (t_112_ = 3.08, *p* = 0.003). There was no significant change in negative mood or general emotional arousal. See Supplementary Table S1 for full descriptive and comparative statistics. Again, the Museum cohort (Miller et al., 2025) did not answer these questions.

As participants in the Digital condition viewed all eight artworks in a randomized order, we checked for possible viewing order effects as these related to specific key appraisal and interest questions. One-way ANOVAs showed no effect of viewing order on ratings of liking (*F*_7, 896_ = 0.586, *p* = 0.768), beauty (*F*_7, 896_ = 0.503, *p* = 0.833), or meaningfulness (*F*_7, 896_ = 1.137, *p* = 0.337) suggesting against possible interaction or general art fatigue effects (Mikuni et al., 2022).

To also consider potential systematic effects, at the individual viewer level in the Digital cohort, where variance in emotions and experience types could be driven by particular answering trends within participants and across artworks, we also examined intraclass correlation coefficients (ICCs). When used to assess a within-participant repeated measures design (as in the Digital cohort), this provides a means of considering whether variance is primarily attributed to within or between-participant factors (Koo and Li, 2016; Pelowski et al., 2024). Scores < 0.50 tend to indicate high within-participant variance and, thus, higher artwork-by-artwork fluctuations; ICCs >0.50, and especially >0.75, suggest trends that are relatively stable within a participant. Here, ICC values were low for all rated items ( ≤ 0.32 for the 16 phenomenal state items; ≤ 0.37 for artwork ratings; see Supplementary Table S2 for all ICC values and confidence intervals).^2^ Given the absence of viewing order effects and the low between-participant variance—which together indicate the within-subjects design is unlikely to meaningfully affect further results, all subsequent analysis were conducted on a trial-level basis, for both the Digital and Museum cohorts.

### Artwork ratings and wellbeing impacts between digital and museum conditions

**Figure 2:**
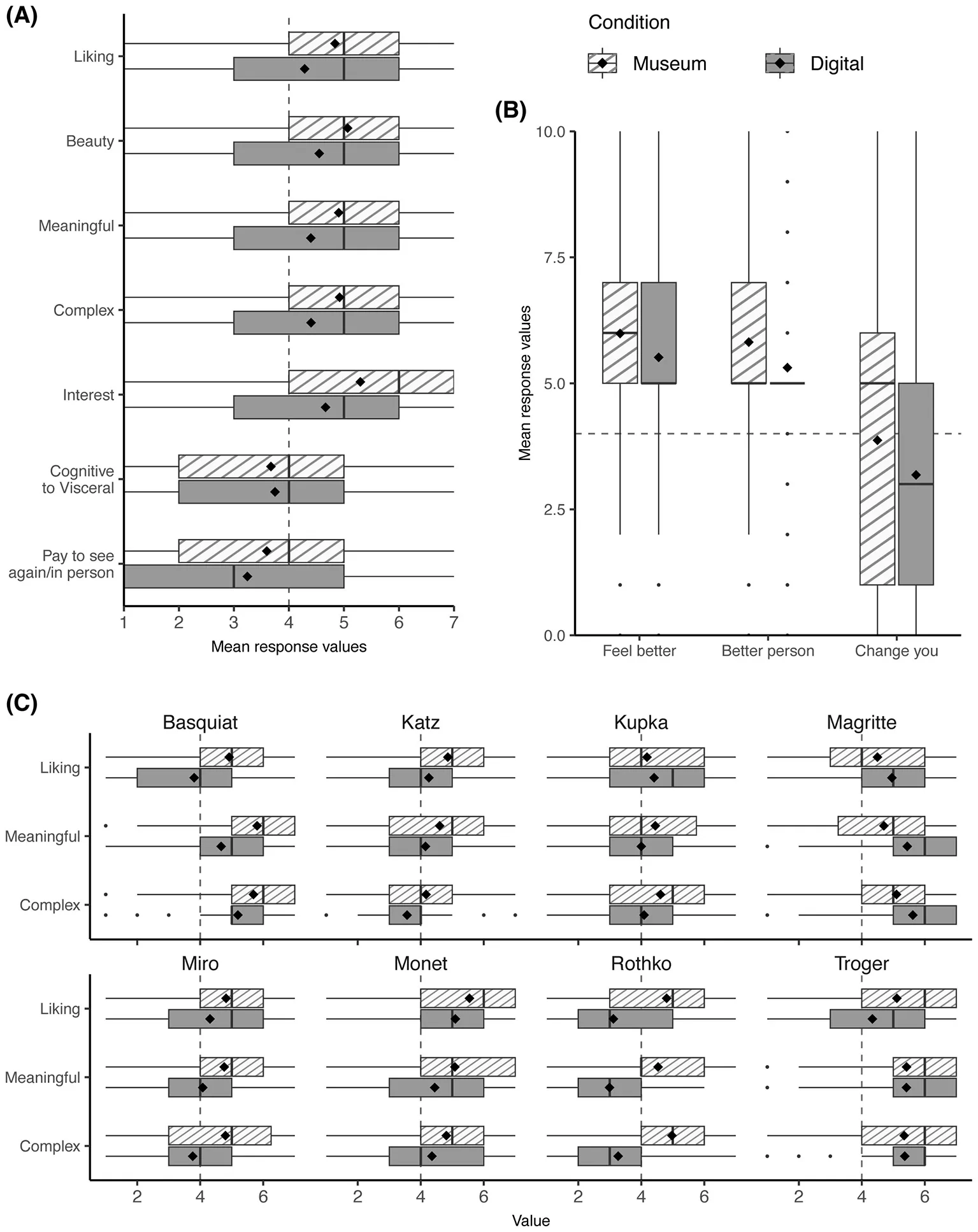
Artwork ratings compared between Museum and Digital conditions and by artwork. For all panels, boxes show groupwise medians and interquartile ranges; large points indicate groupwise mean ratings. White boxes with gray stripes represent ratings by the Museum cohort (*N* = 745); solid gray boxes are those by the Digital cohort (*N* = 904). **(A)** Results of aesthetic judgement ratings (rated on 7 pt. bipolar scales). With the exception of “Cognitive to Visceral,” all variables showed a main effect of condition. Results are combined across levels of artwork. **(B)** Results of impact ratings (rated on 11 pt. bipolar scales). All variables showed a mean effect of condition. Results are combined across levels of artwork. **(C)** Select aesthetic judgement ratings are shown again, now depicting observed interaction effects between condition and artwork. See Supplementary Table S3 for full results of two-way ANOVAs assessing differences in judgement and impact ratings between conditions and artworks.

Descriptive statistics for participants' ratings of the artworks as well as the impact questions are reported, for each condition and for each artwork within each condition, in Supplementary Table S3. We first assessed the main effect of condition, while accounting for a potential interaction with artwork, by conducting a series of two-way ANOVAs with both condition and artwork included as predictors, run separately for each rating item. Results are shown in Supplementary Table S4; select aesthetic judgement ratings between the two conditions are also shown in Figure 2.

For almost all variables, we detected significant main effects of both artwork and condition, as well as significant interactions, after correcting for multiple comparisons (*p* = 0.05/13 analyses = 0.0038).^3^ This included all the artwork ratings (e.g., liking, beauty, meaning, complexity, interest) and some impacts, including one's willingness to pay to see a given artwork again/in person and whether one feels better after their art viewing. Ratings of how detached/cognitive or visceral/emotional one's experience was showed only a main effect of artwork, while ratings of additional impact terms, such as feeling oneself to be a better person and feeling that the experience had changed the viewer, showed only a main effect of condition.

The recurrent main effects of artwork were anticipated, as these varied in their style and content, and generally aligned with the ratings differences found from the original museum study (Miller et al., 2025). Across all variables that showed a significant main effect of condition, participants tended to report higher, or more positive, ratings when viewing an artwork in the museum as opposed to the digital, laboratory condition (see Figures 2A, B; Supplementary Table S4). Looking descriptively to the interactions between condition and artwork and to the potential variance in judgement ratings across the specific artworks, participants tended to rate artworks, with the exception of the Magritte, lower in the Digital condition (Figure 2C). At the same time, some ratings for some artworks (e.g., ratings of liking and willingness to pay to see again/in person for the Kupka and ratings of meaningfulness and complexity for the Troger) showed little between conditions. These interactions suggest that different artworks are more and less affected by the shift in condition, but we generally did not find any easily interpretable descriptive trends to explain these differences.

The effect sizes of both the main and interaction effects were consistently small to moderate across all rated variables. The main effects of artwork typically showed the largest effect on ratings (η^2^ values for significant effects ranging from 0.04 to 0.13; Supplementary Table S4). Effect sizes for the main effects of condition and the observed interactions were small (η^2^ ≤ 0.04 across all variables), suggesting a consistently minimal, albeit significant, impact of condition across all artworks.

### Emotional-phenomenal experience ratings

Descriptive statistics of participants' reports of their felt-experience with each artwork, via the 16-items, broken down between conditions, as well as individual artworks, are shown in Supplementary Table S5. As can be seen, and as expected, we found a quite wide distribution of responses across the artworks, the particular emotion terms, and individual participants. Notably, for most of the terms, across all artworks, we also found that each cohort tended to use all points of the scales given.

**Figure 3:**
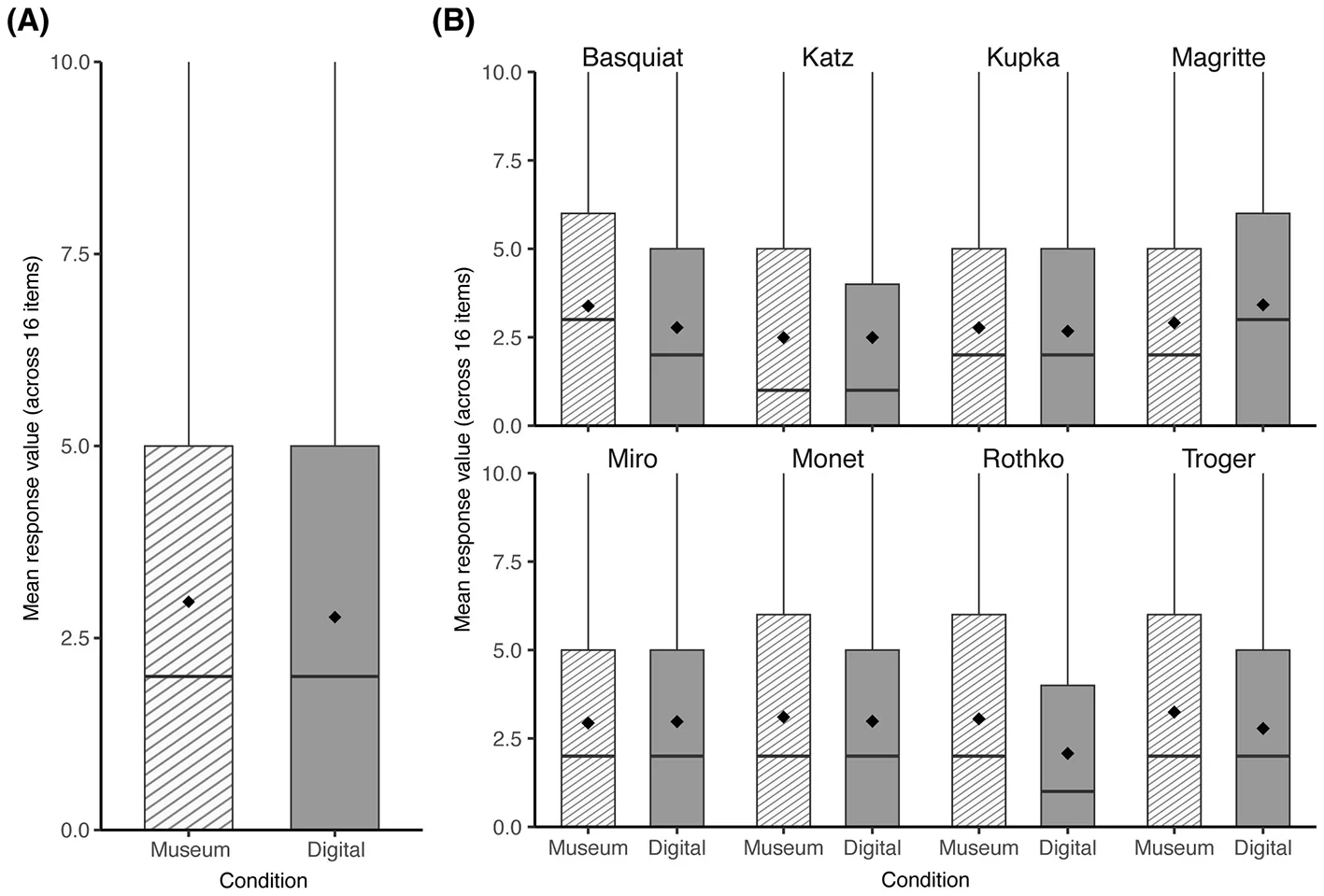
Averaged phenomenal state ratings between Museum and Digital conditions and by artwork. For all panels, boxes show groupwise medians and interquartile ranges; large points indicate groupwise mean ratings. White boxes with gray stripes represent ratings by the Museum cohort (*N* = 745); solid gray boxes are those by the Digital cohort (*N* = 904). Phenomenal state ratings are shown averaged across the 16 included states (rated on 11 pt. bipolar scales) to assess the overall magnitude of reported phenomenal response. **(A)** Averaged phenomenal state ratings between Museum and Digital conditions. A significant main effect of condition is depicted. **(B)** Averaged ratings, now also by individual artwork (*N* = 8), between Museum and Digital conditions. Results showed a significant interaction effect between artwork and condition. See Supplementary Table S5 for full results of the conducted two-way ANOVA.

To begin to assess potential differences between conditions and artworks, we first considered the overall magnitude of reported emotions, averaging across all 16 rated items. A two-way ANOVA (reported in Supplementary Table S6) revealed a significant main effect of condition, where the overall magnitude of reported feeling was significantly higher for the Museum (M = 2.97, SD = 3.08) than the Digital group (M = 2.77, SD = 2.95), though the pairwise comparison showed a negligible effect size (absolute Cohen's *D* = 0.072). This analysis further showed an additional, and expected, main effect of artwork, as well as a significant interaction effect (Figure 3). Pairwise comparisons of estimated means of by-artwork ratings showed a significant effect of condition on the overall magnitude of phenomenal ratings for four of the eight artworks, where the overall magnitudes of reported phenomenal states with the Basquiat, Rothko, and Troger pieces were significantly higher in the Museum. On the other hand, reported feeling was higher in the Digital setting only with the Magritte (Supplementary Table S6).

To further consider possible differences, this time at the level of specific phenomenal items, we conducted a series of two-way ANOVAs for each of the 16 states, examining effects of both condition and artwork (Supplementary Table S7). These did reveal a main effect of artwork for all states, as well as a main effect of condition and interaction effect for many states. There was, however, no clear pattern to interpret these comparisons in detail; see also Supplementary Figure S1.

### Reported phenomenal experience types

We then moved to the main assessment of experience types, based on the reported phenomenal experiences.

#### Assigning experience types by confirmatory latent profile analysis

To assess ExpTypes, we applied a confirmatory latent profile analysis with deterministic constraints in Mplus 8 (CLPA; Weller et al., 2020; Sinha et al., 2021; Muthén and Muthén, n.d.), based on the analysis of Miller et al. (2025).^4^ From their original LPA model, we extracted the standardized mean values and variances of each of the 16 rated phenomenal items for all five profiles. We then input these as starting values, or deterministic constraints, for the CLPA with the participant reports from newly collected Digital cohort. In such a confirmatory analysis, the algorithm does not recalculate what the specific profiles are; it merely assigns the new participants to one of the five pre-defined profile groups.

CLPA model fit was assessed primarily by entropy and average latent class posterior probability (ALCPP) values, as well as consistency of auxiliary results between the new and original analyses (Muthén and Muthén, n.d.; Nylund et al., 2007).^5^ In the context of latent model analyses, entropy is a measure of profile distinction, while ALCPPs are indicators of participant-level profile assignment confidence. For both indices, higher values (i.e., closer to one) indicate higher model fit, with values over 0.8 typically deemed as acceptable; for ALCPPs in particular, values over 0.9 are preferred (Muthén, 2008; Weller et al., 2020). Auxiliary analyses assess group-level differences in secondary variables and are comparable to a Wald's test of mean equality or a one-way analysis of variance (Asparouhov and Muthen, 2014; Muthén and Muthén, n.d.; Nylund et al., 2007).

Through this deterministic CLPA, we assigned each participant report (at the artwork-level) to one of the five experience type profiles identified by Miller et al. (2025)—Harmonious, Novel, Transformative, Disengaged, or Negative. The CLPA solution showed both good differentiation of profiles (entropy = 0.888) and high confidence in individual ExpType assignments (M_ALCPP_ = 0.924, range = 0.895–0.945). Auxiliary analyses showed a similar pattern of relationships between the profiles and judgement/impact ratings as the original museum study (Supplementary Table S8; compared to Supplementary Table S9 of Miller et al., 2025).^6^ These findings supported the sufficient fit of new data to the original five-profile model, as well as consistent character of the profiles between the original and current datasets.

#### Experience type incidence between digital and museum conditions

**Figure 4:**
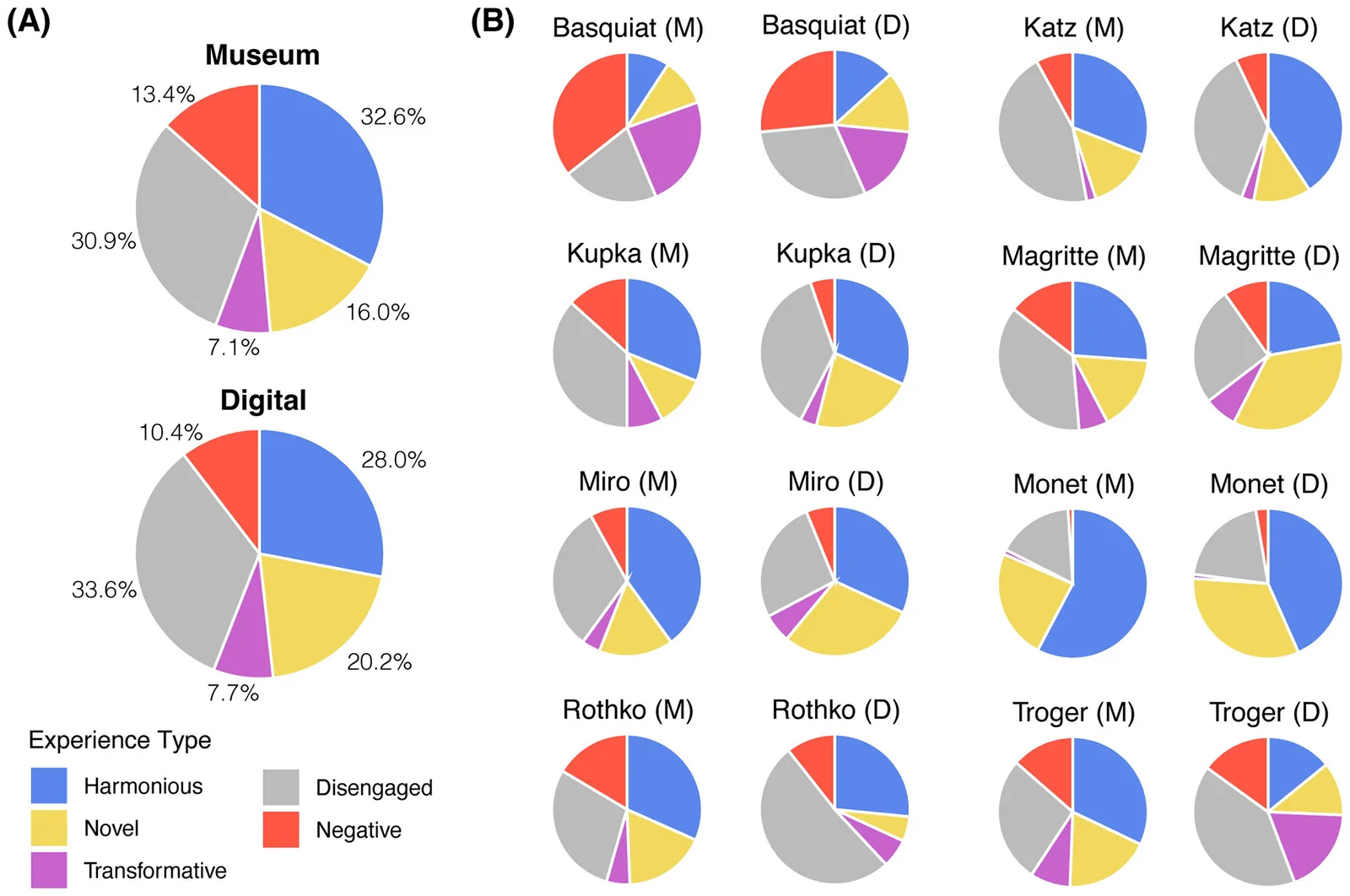
Frequency of experience type Occurrence: between conditions and by artwork. Plots depict the percentage of participants determined to have a particular type of emotional-phenomenal experience, according to a five-profile latent profile analysis. **(A)** Experience type frequencies are shown between conditions (Museum and Digital), across all participants and artworks. **(B)** Frequencies are shown between conditions and artwork. Artworks are indicated by the last name of the artist (see Table 1 for detailed list); next to each artist name, “(M)” indicates experience type frequencies for that artwork found in the Museum condition, “(D)” indicates the Digital condition.

The distributions of ExpTypes, as assigned to experiences reported by both the Museum and Digital cohorts, divided also between individual artworks, are shown in Figure 4. Critically, within the Digital dataset, we found no effect of viewing order on ExpType in the lab-based experiences [χ^2^ (28) = 36.585, *p* = 0.128]; we also found no effect of viewing time [χ^2^ (28) = 8.845, *p* = 0.065]. As can be seen in Figure 4, we found a range of reported experience types between individuals and artworks, with all artworks found to elicit at least one of each experience type in both conditions.

Within the Museum condition, we did find the incidence of ExpType to vary significantly between artworks [χ^2^(28) = 157.240, *p* < 0.001, Cramer's *V* = 0.230], as expected based on our selection from the original study. A similar significant relationship was found within reported experiences from the Digital cohort [χ^2^(28) = 193.850, *p* < 0.001, Cramer's *V* = 0.232], indicating a similar rate of artwork-level variance in ExpType distribution for the two conditions. At the same time, a separate chi-squared analysis revealed a significant difference in overall ExpType distribution between the digital and museum conditions [collectively across all eight artworks; (χ^2^ (4) = 11.329, *p* = 0.0231)]. The effect size, however, suggested only a weak association (Cramer's *V* of 0.083). In general, this appeared to be driven by a lower incidence of Harmonious and Negative experiences, as well as a higher incidence of Novel and Disengaged experiences, in the Digital condition, as compared to the Museum condition. There was no notable change in the rate of Transformative experiences.

As noted above, our Digital and Museum groups did differ slightly in regard to both age and gender distribution. Auxiliary analyses previously conducted for the original museum study suggested that age—but, notably, not gender—did show some variance across experience types. Specifically, those individuals who reported Negative experiences tended to be younger than those who reported Harmonious, Novel, or Disengaged experiences (see Miller et al., 2025). We repeated these auxiliary analyses with data from the Digital group and found identical results (Supplementary Table S8). Gender again showed no relationship with experience type distribution. Age did vary significantly between the ExpType groups, and pairwise analyses showed the same pattern as was seen in the Museum cohort (see Miller et al., 2025).

To consider possible differences in experience type incidence between the two conditions as a function of the different age distributions, we extracted age distribution-matched subsets for each of the Digital (*n* = 578, M = 29.29, SD = 9.43, range = 18–64) and Museum groups (*n* = 578, M = 29.57, SD = 9.90, range = 18–67). A chi-squared analysis of the ExpType distribution in the subset data again showed a significant difference in ExpType frequency between the two conditions [χ^2^ (4) = 11.420, *p* = 0.022]. This effect was comparable to that from the same analysis of the original (i.e., whole) dataset, which suggests that the difference in ExpType incidence is not a function of different aged samples (see also Supplementary Figure S3). A subsequent multinomial regression of dataset (original vs. subset), condition, and artwork showed no significant effect of dataset on ExpType distribution and no interactions between dataset and condition and/or artwork (see Supplementary Table S9). Thus, these analyses supported the argument that the different age distributions were not drivers of the noted difference in ExpType frequency between conditions.

#### Artwork ratings by both condition and experience type

Descriptive statistics of aesthetic judgement and impact measures are reported, broken down by ExpType, in Supplementary Table S10. We next repeated the series of ANOVAs across judgement and impact ratings, now including ExpType as a third predictor. Results are reported in Supplementary Table S11. These analyses replicated the earlier main effects of condition and artwork, as well as their interaction effect, again showing that ratings were, in general, more positive in the museum and that this effect of condition was stronger for some artworks than others. We also observed a main effect of ExpType for all ratings, with moderate to large effect sizes (η^2^ values ranged from 0.04 to 0.27). Results were consistent with findings from the original, larger museum study (Miller et al., 2025) and mimicked those of the earlier reported auxiliary analyses (Supplementary Table S8)—where ratings of harmonious and novel experiences tended to be highest, and disengaged and negative experiences were typically rated lowest. Importantly, no analyses showed an interaction effect between ExpType and artwork, again consistent with Miller et al. (2025) and supporting the stability of the profiles across stimuli.

**Figure 5:**
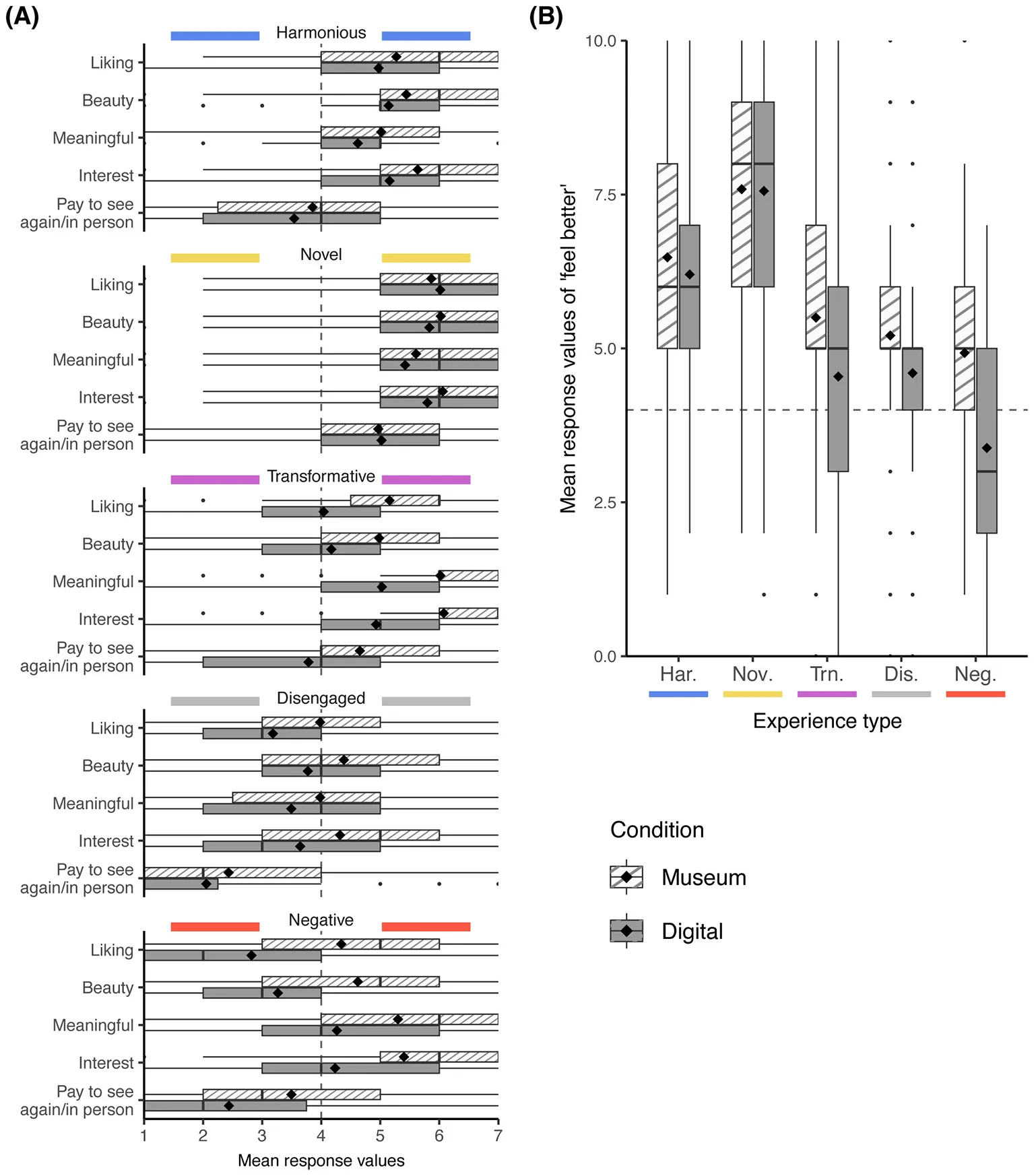
Artwork ratings compared between experience types and conditions. For all panels, boxes show groupwise medians and interquartile ranges; large points indicate groupwise mean ratings. White boxes with gray stripes represent ratings by the Museum cohort (*N* = 745); solid gray boxes are those by the Digital cohort (*N* = 904). Experience types are labeled along the x-axis by name, as well as indicated visually by color. For all variables shown in both panels, a three-way ANOVA between experience type, condition, and artwork indicated a significant interaction between experience type and condition. **(A)** Aesthetic judgement ratings (rated on 7 pt. bipolar scales) by experience type and condition. **(B)** Impact rating, for reports of feeling better after viewing an artwork (rated on 11 pt. bipolar scales) by experience type and condition. See Table 3 for pairwise conditions for depicted variables; see Supplementary Table S10 for full results of the conducted three-way ANOVAs.

Several ratings showed an interaction between condition and ExpType (liking, beauty, meaning, complexity, interest, reports of feeling better; Figure 5; Supplementary Table S11). Based on the observed interaction between condition and artwork, this was expected as the frequency of ExpType also varies across artworks. We conducted further pairwise comparisons for these variables to clarify the details of the observed significant interactions, specifically looking at the effect of condition within each ExpType group (Table 3). Results showed a significant effect of condition on all ratings for Transformative, Disengaged, and Negative ExpTypes. Harmonious experiences showed an effect of condition on ratings of meaningfulness, complexity, interest, and reports of feeling better after the art-viewing. Ratings of liking and beauty did not differ between conditions for Harmonious experiences, and none of the ratings differed for Novel experiences.

**Table 3:** Pairwise comparisons of museum & digital cohort ratings between experience types.

Variable	ExpType	estimate	SE	*df*	t.ratio	*p*-value	Effect.size
Liking	Harmonious	0.214	0.152	1,556	1.409	0.159	0.143
	Novel	−0.154	0.196	1,556	−0.788	0.431	−0.103
	Transformative	1.483	0.403	1,556	3.678	0.000^***^	0.988
	Disengaged	0.792	0.139	1,556	5.689	0.000^***^	0.528
	Negative	1.150	0.304	1,556	3.780	0.000^***^	0.766
Beauty	Harmonious	0.252	0.136	1,566	1.844	0.065	0.186
	Novel	0.121	0.176	1,566	0.684	0.494	0.089
	Transformative	1.219	0.362	1,566	3.369	0.001^***^	0.900
	Disengaged	0.575	0.125	1,566	4.600	0.000^***^	0.425
	Negative	0.931	0.275	1,566	3.390	0.001^***^	0.687
Meaning	Harmonious	0.308	0.145	1,566	2.119	0.034^*^	0.213
	Novel	0.356	0.188	1,566	1.895	0.058	0.247
	Transformative	1.313	0.385	1,566	3.407	0.001^***^	0.910
	Disengaged	0.507	0.133	1,566	3.798	0.000^***^	0.351
	Negative	0.864	0.292	1,566	2.956	0.003^**^	0.599
Interest	Harmonious	0.352	0.150	1,567	2.345	0.019^*^	0.236
	Novel	0.342	0.194	1,567	1.763	0.078	0.230
	Transformative	1.435	0.398	1,567	3.605	0.000^***^	0.963
	Disengaged	0.662	0.138	1,567	4.813	0.000^***^	0.444
	Negative	0.854	0.302	1,567	2.827	0.005^**^	0.573
Pay to see again/in person	Harmonious	0.155	0.164	1,556	0.946	0.344	0.096
	Novel	0.056	0.213	1,556	0.262	0.793	0.034
	Transformative	0.897	0.433	1,556	2.069	0.039^*^	0.553
	Disengaged	0.426	0.150	1,556	2.847	0.004^**^	0.263
	Negative	0.898	0.329	1,556	2.731	0.006^**^	0.554
Feel better	Harmonious	0.147	0.167	1,553	0.877	0.381	0.088
	Novel	0.253	0.217	1,553	1.169	0.243	0.153
	Transformative	1.178	0.443	1,553	2.660	0.008^**^	0.711
	Disengaged	0.522	0.153	1,553	3.406	0.001^***^	0.315
	Negative	1.830	0.337	1,553	5.424	0.000^***^	1.105

A series of three-way ANOVAs assessed differences in artwork ratings across conditions, experience types, and artworks. Results indicated a significant interaction between condition and ExpType for six variables. Pairwise comparisons of these interactions are reported, averaged across levels of artwork. See Supplementary Table S10 for full reporting of the ANOVAs. *p < 0.05. **p < 0.01. ***p < 0.001.

**Figure 6:**
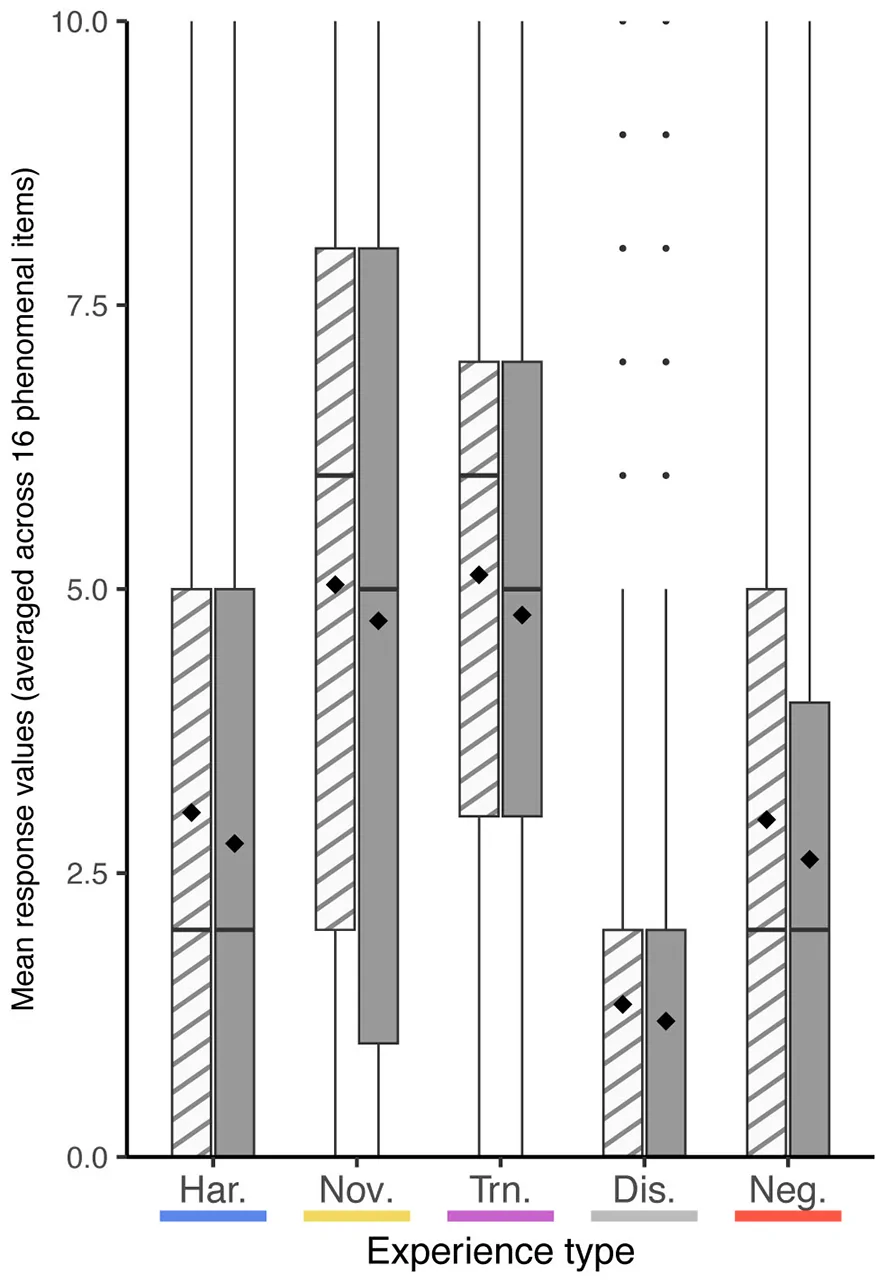
Averaged phenomenal state ratings between experience types and conditions. For all panels, boxes show groupwise medians and interquartile ranges; large points indicate groupwise mean ratings. White boxes with gray stripes represent ratings by the Museum cohort (*N* = 745); solid gray boxes are those by the Digital cohort (*N* = 904). Experience types are labeled along the x-axis by name, as well as indicated visually by color. Phenomenal state ratings are shown averaged across the 16 included states (rated on 11 pt. bipolar scales) to assess the overall magnitude of reported phenomenal response. A three-way ANOVA showed significant main effects for experience type, condition, and artwork. There was no interaction effect between experience type and condition, as depicted in the Figure. See Table 4 for full results of the conducted three-way ANOVA; see Supplementary Figure S3 for visualization by artwork, in addition to experience type and condition.

We also looked back to the overall and individual ratings of the phenomenal states between conditions, now also incorporating ExpType; average phenomenal ratings (overall and by item, broken down by ExpType and condition) are reported in Supplementary Table S12. We repeated the earlier analysis of the magnitude of overall phenomenal state ratings, now as a three-way ANOVA, integrating condition, artwork, and ExpType (Table 4). Results showed a main effect of each independent variable, with the patterns of findings consistent with previously reported analyses (Figure 3; Supplementary Table S6). We found no interaction between condition and ExpType or between condition and artwork, indicating that the main effect of condition (overall magnitude of phenomenal ratings as higher in the Museum) was independently consistent across ExpTypes and artworks (Figure 6). We did find an interaction between artwork and ExpType, indicating that the magnitude of overall response for each artwork depended on individual ExpType (Supplementary Figure S3). Pairwise comparisons within each artwork showed that the magnitude of ratings generally differ between ExpType groups (Supplementary Table S13A); however, comparisons within each ExpType showed that the magnitude of response did *not* differ between artworks (Supplementary Table S13B). There was no significant three-way interaction.

**Table 4:** Three-way ANOVA results of overall magnitude of phenomenal state ratings.

Effect	*df*	Sum Sq	Mean Sq	*F*-value	Pr(>F)
Condition	1	261	261	36.46	< 0.001^***^
Artwork	7	1,567	224	31.25	< 0.001^***^
ExpType	4	48,113	12,028	1,679.28	< 0.001^***^
Condition: Artwork	7	86	12	1.72	0.10
Condition: ExpType	4	41	10	1.42	0.22
Artwork: ExpType	28	462	17	2.31	< 0.001^***^
Condition: Artwork: ExpType	28	220	8	1.10	0.33
Residuals	26,297	188,360	7		

Overall magnitude refers to ratings averaged across all 16 phenomenal states (each rated on 11 pt. scale, 0–10; see Supplementary Table S4 for summary statistics). ***p < 0.001.

### Further insights into digital experiences

Finally, we assessed the additional questions responded to only by the Digital group. We first assessed reported sense of immersion, which, like the ratings above (i.e., liking, beauty, etc.), was assessed following each artwork engagement. An auxiliary analysis (Supplementary Table S8) identified a significant difference in ratings of immersion between the ExpType groups between artworks (χ^2^ = 495.44, *p* < 0.001). Pairwise comparisons showed a significant difference between all ExpTypes (at *p* ≤ 0.002), with the exception of the Harmonious and Transformative types; Novel experiences were rated as the most immersive, followed by Harmonious/Transformative, then Negative, and Disengaged experiences were rated as the least immersive.

At the end of the study, participants in the Digital condition also reflected on how their experiences in the study compared to their previous museum experiences; ratings for all items are reported in Table 5. In general, participants did rate their experiences as relatively different to those they had previously had in museums (M = 3.06 out of 7, SD = 1.33; *1* = *completely different, 7* = *completely the same*). Experiences were further rated as less visually pleasing (M = 2.79, SD = 1.28), emotional (M = 2.81, SD = 1.41), and immersive (M = 2.72, SD = 1.52) than those in museums. Finally, participants also rated the extent to which they thought that certain aspects had contributed to what made their experiences in the lab different from those in museums (1 = Not at all, 7 = Very much). All eight factors were rated as relevant (with mean ratings above the midpoint); artwork size, atmosphere of the museum, and seeing the real artwork were rated the most highly.

**Table 5:** Perceived similarity/difference of digital to museum experiences.

Item	*M*	*SD*
To what extent were your experiences today similar to those you have had in museums?	3.06	1.33
*Compared to past experiences you have had in museums, was your experience today as …*		
As visually pleasing	2.79	1.28
As emotional	2.81	1.41
As immersive	2.72	1.52
*To what extent do you think each of the factors below contributed to any differences between your experiences here today and past experiences you have had in museums?*		
The atmosphere of the museum	5.62	1.36
The architecture of the museum	4.87	1.85
The social environment	4.36	2.06
A sense of immersion	5.50	1.43
Being able to move freely	5.41	1.61
The size of the artwork(s)	5.81	1.46
The visual quality of the artwork(s)	5.34	1.73
Being able to see the real artwork	5.55	1.67

Items were responded to by the Digital cohort only. All items were rated on 7-point scales. The first item was rated on a bipolar scale [1 = “Not at all the same (completely different),” 7 = “Completely the same (not at all different)”]. The next three items were also rated on bipolar scales (1 = “Significantly less so,” 4 = “Exactly the same,” 7 = “Significantly more so”). The remaining items were rated from 1 = “Not at all” to 7 = “Very much.”

## Discussion

This study investigated potential differences between engagements with museum/original and digital art, contributing to a growing collection of literature that considers the effects of context and genuineness. We compared reported experiences had by cohorts of individuals as they encountered a selection of genuine artworks from important masters and institutions in Europe to a new collection of engagements with the same works of art, this time viewed as high-quality digital replicas within a traditional laboratory study design. Our assessment targeted both commonly assessed hedonic and aesthetic judgement ratings as well as the more global and nuanced phenomenal experience, by employing a new tool developed for comparative visual art study (Miller et al., 2025). Especially these emotional, embodied, and broadly phenomenal aspects of an art engagement were argued in this and previous studies to perhaps provide a better candidate for detecting salient differences between museum/digital modalities, but had not yet been systematically empirically considered.

In turn, our findings did both support and extend beyond previous research. First, when looking to commonly assessed rating items (e.g., liking, beauty, meaningfulness), and in parallel to previous studies (Darda et al., 2025; Estrada Gonzalez, 2023; Specker et al., 2017), analyses showed that ratings did tend to be generally higher in the Museum than in the Digital condition. However, identified differences were consistently accompanied by negligible to small effect sizes. Across a number of variables, we found small interaction effects between condition and artwork, suggesting that different artworks are more or less affected by the change in condition. Primary results from this first level of analysis were consistent with previous empirical studies that found only small differences in participant evaluations of artworks between museum and lab studies (Darda et al., 2025; Specker et al., 2023b).

At the same time, participants in the Digital condition did recognize and report their experiences as different than those they had previously had in museums. Despite only small to negligible differences in ratings of individual artworks, participants seem to remain aware of an experiential discrepancy between art engagements under these two conditions. This self-reported awareness may lend support to, for example, the “facsimile accommodation hypothesis” (Locher et al., 2001). Participants seem to have compensated in the lab setting when evaluating individual reproductions while still being cognizant that the broader experience differed from that of an art encounter within a museum. Participants further rated their art experiences in the study as not only different but less visually pleasing, less emotional, and less immersive than prior museum experiences—perhaps reflecting and supporting the view that something is “lost” in the translation of an artistic object from physical to digital (Benjamin, 1936).

The persistence of reports that the experiences of museum originals and digital reproductions *seem* different aligns with arguments from previous researchers that assessment methods should be extended beyond simple aesthetic judgements, as these seem unable to reveal some of the differences that are felt and reported (Darda et al., 2025; Specker et al., 2023b).

### Extending assessment methods to emotional-phenomenal experience

When next considering overall phenomenal ratings (i.e., averaged feelings across all 16 phenomenal items) across all artworks and conditions, we again found consistent, but small, significant effects of condition, where phenomenal ratings tended to be higher in the Museum. These effects varied across stimuli, as overall magnitude of phenomenal ratings did not differ for certain artworks (Katz, Kupka, Miro, Monet), while others (Basquiat, Rothko, Troger) were rated higher in the Museum; only one artwork (Magritte) was rated more highly in the Digital condition. This interaction paralleled those observed with the artwork ratings, further supporting that different artworks varied in terms of the strength and direction of condition-based effects. Given the range of artwork styles and valence of depicted content, as well as the variance in overall magnitude of response between artworks, this interaction may be dependent on variation in evoked emotional response (Bruns et al., 2023; Menninghaus et al., 2019; Miller et al., 2024; Pelowski et al., 2017b).

This paper went further by also applying the latent profile model procedures—as specified in the museum collection of Miller et al. (2025)—to ratings of the 16 phenomenal states from the Digital cohort. Model fit indices supported the application of the latent profile model and yielded high confidence in individual-level ExpType assignments. The resulting experience types as assigned at the individual viewer level, in turn, suggested that we could indeed detect a range of responses to each work of art across both conditions. Surprisingly however, the overall distribution of ExpTypes differed only slightly between the Museum and Digital groups. From the Museum to the Digital group, reports showed a slight reduction in the proportion of Harmonious and Negative experiences, alongside a slight increase in Novel and Disengaged experiences.

Given the overlap and distinction between the Novel and Harmonious experiences—specifically that both present with high levels of positive affect, but only the Novel profile shows high ratings of (meta)cognitive states—it may be that the Digital condition prompted more cognitive, and less purely affective, engagement, thus resulting in a higher proportion of Novel experiences. While museums scaffold an embodied and affectively grounded experience (Pelowski and Akiba, 2011; Brieber et al., 2014; Malarux, 1974), digital contexts are suggested to loosen the typical protocols and expectations that govern traditional art viewing (Duncan, 1995; O'Doherty, 1986). This may further encourage more interpretive openness (Cameron, 2007) and an altered reliance on metacognitive (as opposed to affective) engagement that helps explain why digital encounters might be more frequently categorized as Novel than Harmonious, despite the relatively similar experience types across conditions.

A similar parallel may be drawn between Disengaged and Negative experiences. The reduced incidence of Negative experiences in the Digital condition may indicate a greater ability to maintain distance from an artwork that has the potential to challenge the viewer or cause discomfort, with viewers instead reporting feeling bored (i.e., Disengaged). This is apparent in, for example, reported experiences of the Basquiat, which were 35.6% Negative and 20.1% Disengaged in the Museum, but 26.5% Negative and 30% Disengaged in the Digital condition. Meeting a challenging artwork in the museum—perhaps by virtue of being presented with the genuine artwork (Newman and Bloom, 2012; Specker et al., 2023a) or one's individual and social expectations of the museum (Brieber et al., 2015b; Falk and Dierking, 2016; Pelowski et al., 2017a)—may encourage a deeper, more sustained engagement and foster a viewer's willingness to actually experience the negative response.

### Influence of experience type across conditions

Beyond revealing changes in ExpType proportion between conditions, by assigning ExpTypes to individual reports, we were able to treat ExpType as a third independent variable to see if/how this interacted with condition and/or artwork to reveal nuances not previously observed. As expected, we found moderate to large main effects of ExpType across variables. This effect was consistently larger than those of condition or artwork, suggesting that how a participants' experience *feels to them*, according to their self-reported phenomenal experience, was the most impactful driver of judgement ratings, regardless of the specific artwork or viewing condition. We found no significant interactions between ExpType and artwork, which is consistent with the idea that, while frequency of occurrence will vary, the character of the profiles, with respect to how they are associated with judgement ratings, will be consistent across artworks.

There was, however, a recurrent, significant interaction between ExpType and condition for a number of rating variables, including liking, beauty, meaningfulness, and reports of feeling better after engaging with the artwork. Judgement ratings were significantly diminished in the Digital condition primarily for the Negative, Transformative, and Disengaged ExpTypes, with much larger effects than those seen when combined across all engagements. There was no difference in any judgement ratings between conditions for those who reported Novel, and very few for Harmonious, experiences. This strong interaction effect may shed light on why previous studies—including our first set of analyses here, which considered condition alone, thus collapsing all types of reported felt-experience together—have revealed only small effects of context on aesthetic judgements.

These findings that suggest negative or challenging experiences in particular require the contextual support of museum in order to be integrated into meaningful experience outcomes (Darda et al., 2025; Leder et al., 2004) Museums and institutions—which are respected by society at large and hailed as pillars of culture, history, and social engagement (Cameron, 2007; Duncan, 1995)— provide not only symbolic but also embodied scaffolding that allows viewers to process uncomfortable feelings, such as confrontation or ambiguity, in a literal safe space. In comparison, the laboratory settings or digital environments do not guide the viewers' experience with such supportive frames, or other social/performance expectations. Thus, a potentially negative or challenging experience may be detached from before it reaches this point, as it may be easier to quickly click or scroll passed digital content than to walk away from a difficult work that is physically presented in front of them and socially framed within the museum.

As noted previously, the overall magnitude of phenomenal ratings—across all artworks–was higher in the Museum than the Digital experiences; this further showed an interaction with artwork, where some artworks were more affected (i.e., showed more of a difference between the conditions) than others. The later three-way analysis revealed this dampening effect of the Digital condition to be consistent across each of the five ExpTypes (i.e., no significant interaction between condition & ExpType). Additionally, the previously observed interaction between condition and artwork was no longer significant; instead, we saw an interaction between ExpType and artwork emerge. Specific pairwise comparisons showed that this interaction was due to differences in magnitude for the ExpType groups within a given artwork. Within an ExpType group, the average magnitude of response did not vary between artworks, suggesting that the observed variance in the overall magnitude of phenomenal reports between artworks was being driven by inherent differences in the magnitude of reports for each of the ExpTypes, as well as the variance in ExpType proportion between artworks. Together these findings suggest that what initially presented as an effect of artwork—in the initial analyses of only condition and artwork—in the original analysis may instead be an effect of ExpType, and specifically a side effect of combining reports within an artwork across ExpTypes, therefore collapsing the variance between subgroups.

### Implications for theory, applications, and stakeholders

Our findings echo broader theoretical perspectives on how setting might modulate aesthetic experience, with art-critical arguments and some prior empirical work suggesting that museum environments provide something that laboratories cannot—sensory, spatial, and cultural affordances that amplify emotional resonance (Brieber et al., 2015b; Pelowski et al., 2017a). In comparison, digital viewings in a laboratory setting were shown to soften the magnitude of averaged reported phenomenal states, which is in line with the idea technological reproduction can diminish the aura of an artwork (Benjamin, 1936). This reduction in intensity of emotional response was small, suggesting that a digital engagement does not result in a total alteration or disruption of felt response, but rather a subtle decrease in the magnitude of force of the experience.

Digital initiatives have been enthusiastically adopted and promoted by museums as tools for expanding access, yet as we have suggested their actual impact remains uneven and poorly understood (Cao, 2024). While the underlying structure of art experience appears stable across the Museum and Digital conditions, this framework of identifying and assessing specific varieties of experience offers curators, museums, and art professionals an informed digital strategy, as we find that different artworks and experience types may vary in their suitability for use in digital contexts. Artworks that translate well into digital contexts—such as those that tend to evoke Harmonious or Novel experiences—should be at the forefront of digital engagement efforts, whereas artworks that more frequently produce Transformative or Negative profiles appear to require the full support of the onsite environment. The consistent reduction in reported magnitude of felt phenomenal aspects, and especially the significantly lower ratings of challenging artworks, suggests that digital formats might not be able to replace the socially scaffolded and embodied script of the art encounter in a contemporary museum setting (Brinkmann et al., 2020; Kühnapfel et al., 2024). This work finds that, rather than a challenge to the traditional museum, digital presentations may be used as an extension to museum collections which increase the likelihood of arts engagement in larger cohorts of viewers (Cameron, 2007).

## Limitations and future research

This study had the unique ability to work with real museum artworks as well as high quality digital reproductions of said originals. However, it included a central limitation in the confounding of context and genuineness, an issue highlighted by previous researchers (Specker et al., 2023b). Here we actively and knowingly applied this confound by comparing the two most dominant and opposite paradigms employed in empirical art studies: original artworks in the museum and digital reproductions on a PC in the laboratory. As such, we are unable to explicitly attribute our results specifically to the authenticity of the object or the setting in which it was encountered, nor can we aid in teasing apart the likely complex relationship between context and genuineness. This is particularly true given the multifaceted nature of each of these aspects in an artwork encounter. The museum and lab settings inherently differ in their architecture, social environment, and capacities for physical movement, while original artworks and digital reproductions not only differ in material but also (typically) in visual quality and image size (Pelowski et al., 2017a). Participants in the Digital cohort specifically rated each of these factors as being relevant to why their art experiences during the study differed from those they had previously had in museums. We suggest that further research introduce intermediate conditions, such as virtual galleries or displaying both original and (full-sized) digitized artworks in both museum and laboratory settings. Such designs would aid in untangling the authenticity of an artwork from the environmental framing so that each can independently be understood to influence aesthetic experiences and reported felt emotions.

Furthermore, largely due to independent pragmatic constraints around the collection of each dataset, the Museum cohort completed a single artwork-viewing experience, whereas the Digital cohort viewed multiple artworks and completed repeated measures. As this inconsistency in dataset structures as atypical, we considered the potential of both within and between-participant effects of the repeated measures design on ratings from the Digital cohort. Low ICC values indicated that the variance in ratings observed with in the Digital cohort was primarily due to within-participant factors. Additionally, no effects of viewing order were found on ratings or ExpType frequencies, suggesting that the observed within-participant variance was not influenced by the repeated measures structure, but by the different artworks presented. Taken with the observed effect sizes in the main analyses (particularly the large effects of ExpType on aesthetic ratings), these analyses support the argument that the repeated measures design of the Digital cohorts did not unduly influence the findings presented. That said, we encourage future research to consider isolating individual artworks in the laboratory and/or having museum visitors view multiple artworks to avoid such statistical concerns.

Finally, the accessibility and demands of working with museum visitors in an onsite recruitment manner and then a replication with paid participants may have led to differences between our two groups that warrant consideration. While the cohorts did not differ significantly on demographic variables (with the exception of age, which was shown to not be driving differences in ExpType frequency between conditions), it should be noted that the difference between spontaneous visitors who opted to participate voluntarily (Museum cohort) and participants who willingly preregistered with advanced knowledge of participation (Digital cohort) may reflect dispositions that are vulnerable to shifts in outcomes. Previous literature has established that different recruitment methods lead to sample-level differences in museum visit motivations, and potentially expectations, both of which have been shown to affect individual experience (Brida et al., 2016; Mastandrea et al., 2007; Tröndle et al., 2014). It is possible that museum visitors might arrive with motivations associated with exploration, learning, and leisure, whereas laboratory goers might arrive with a job-focused or evaluative mindset.

## Data Availability

The raw data supporting the conclusions of this article will be made available upon request to the corresponding author.

## Footnotes

1 For the Museum group, this item was phrased as “to see this artwork again.” For the Digital group, the English phrasing was rephrased to “to see this artwork in person” as intended; the German translation remained phrased as “to see this artwork again.”

2 Estimates and confidence intervals were calculated in R (R Core Team, 2026) using the ICCDesign package (Liu et al., 2026), based on a two-way random effects model with single measurement and absolute agreement (Koo and Li, 2016).

3 As we are repeating this analysis across 13 variables in a series of exploratory analyses, we do not conduct or report specific pairwise comparisons for all analyses. Full ANOVA model results are reported in Supplementary Table S4; select results are depicted in Figure 2.

4 Note, in their original study, Miller et al. (2025) conducted a standard, exploratory LPA with a dataset of *N* = 2,747 participant responses across 31 artworks and identified a five-profile solution, where each profile represented a distinct type of art experience with a unique phenomenal character. This was further contrasted against competing two- to seven-profile solutions that did not show as high a fit across a range of model fit indices.

5 Exploratory LPA models are typically assessed by standard fit indices (AIC, BIC, LL, etc.), however these indices are most valuable in comparing the fit of different solutions and selecting a model for further analysis (i.e., a four- vs. five-profile solution). As the CLPA produces only one, constrained model based on the initial LPA, the raw index values are not independently interpretable (Nylund et al., 2007; Weller et al., 2020).

6 Note also that for Miller et al. (2025) all aux. analyses were done across all 31 artworks included in the original data collection, not restricted to the 8 artworks included in the present study.
