# Supplementary material for: Is there any (emotional) difference between seeing art in the museum vs. in the lab? A comparative study of phenomenal art experiences of original artworks and digital reproductions
Source: Front Psychol. 2026 Sep 8;17:1766700. doi: 10.3389/fpsyg.2026.1766700 (PMC13595691; doi:10.3389/fpsyg.2026.1766700)
Supplement: Supplementary file 1 [file Supplementary_file_1.docx]

**Supplementary Materials**

**Figures.**

**Supplementary Figure S1.** Phenomenal state ratings between Museum and Digital conditions, by individual response item.

*Note.* Boxes show groupwise medians and interquartile ranges; large points indicate groupwise mean ratings. White boxes with gray stripes represent ratings by the Museum cohort (N = 745); solid gray boxes are those by the Digital cohort (N = 904). Results are combined across levels of artwork. See Supplementary Table S7 for results of a series of two-way ANOVAs assessing differences in phenomenal state ratings (by individual item) between conditions and artworks. Analyses indicated a main effect of condition for all items, except for ‘a transformation,’ ‘change in myself,’ ‘active engagement,’ ‘boredom,’ and ‘shock,’ when corrected for multiple comparisons (p = 0.005 / 16 analyses = 0.0031).

**Supplementary Figure S2.** Frequency of experience type occurrence between conditions, in age-matched subsets.

*Note.* Plots depict the percentage of participants determined to have a particular type of emotional-phenomenal experience, according to a five-profile latent profile analysis, in age-matched subsets of the Museum (n = 578; M_age_ = 29.57, sd = 9.90) and Digital (n = 578; M_age_ = 29.29, sd = 9.43) cohorts. See Figure 4 for frequencies between conditions in the full dataset.

**Supplementary Figure S3.** Overall phenom ratings by ExpType, setting, and artwork

*Note.* Boxes show groupwise medians and interquartile ranges; large points indicate groupwise mean ratings. White boxes with gray stripes represent ratings by the Museum cohort (N = 745); solid gray boxes are those by the Digital cohort (N = 904). Phenomenal state ratings are shown averaged across the 16 included states (rated on 11 pt. bipolar scales) to assess the overall magnitude of reported phenomenal response. Responses are shown between levels of experience type (x-axis labels, also indicated by color) and artwork (panels). See Table 4 for results of a three-way ANOVA assessing differences in overall magnitude of phenomenal state ratings (averaged across 16 items) between conditions, experience types, and artworks. See Supplementary Table S13 for pairwise comparisons between conditions at each level of experience type (averaged across levels of artwork; S13A), and at each level of artwork (averaged across levels of experience type; S13B).

**Supplementary Materials**

**Tables.**

**Supplementary Table S1.** Mood ratings (Digital cohort only).

*Note.* Mood was reported by the Digital cohort only. ‘Pre’ ratings were given following all demographic questions, prior to viewing the first artwork; ‘post’ ratings were given immediately after ratings of the eighth (i.e., final) artwork. Change scores were calculated as post – pre, with positive values indicating an increase in that scale from the start to end of the study. Statistical comparisons were conducted as paired-samples t-tests.

| Scale | | Rating | | Pre-Post Comparisons | |
| --- | --- | --- | --- | --- | --- |
|  |  | *M* | *SD* | *t*-statistic (*df*) | Significance (p) |
| Positive mood | | | | 3.08 (112) | 0.0026 ** |
|  | Pre | 5.31 | 1.06 |  |  |
|  | Post | 4.98 | 1.16 |  |  |
|  | Change | -0.33 | 1.13 |  |  |
| Negative mood | | | | -1.07 (111) | 0.28 |
|  | Pre | 2.16 | 1.23 |  |  |
|  | Post | 2.28 | 1.22 |  |  |
|  | Change | 0.13 | 1.23 |  |  |
| General emotional arousal | | | | -0.99 (112) | 0.32 |
|  | Pre | 4.43 | 1.26 |  |  |
|  | Post | 4.56 | 1.38 |  |  |
|  | Change | 0.12 | 1.33 |  |  |

**Supplementary Table S2.** Intraclass correlation coefficients (ICCs; Digital cohort).

*Note.* ICC values calculated for phenomenal state and appraisal/artwork ratings by the Digital cohort. Results are based on two-way random effects models with single measurement and absolute agreement. Values under 0.5 indicate poor reliability, i.e., greater attribution of variance to within-participant factors (Koo & Li, 2016).

| Variable | ICC | 95% CI |
| --- | --- | --- |
| Phenomenal State |  |  |
| Love | 0.25 | [0.18, 0.33] |
| Happiness | 0.18 | [0.11, 0.26] |
| Playfulness | 0.25 | [0.15, 0.36] |
| Pleasure | 0.17 | [0.10, 0.25] |
| Sense of beauty | 0.20 | [0.13, 0.28] |
| Insight | 0.32 | [0.25, 0.41] |
| Moved | 0.22 | [0.16, 0.31] |
| Transformation | 0.31 | [0.23, 0.39] |
| Change in Myself | 0.30 | [0.23, 0.39] |
| Thrilled | 0.22 | [0.15, 0.30] |
| Active engagement | 0.32 | [0.25, 0.41] |
| Boredom | 0.17 | [0.11, 0.25] |
| Shock | 0.26 | [0.18, 0.36] |
| Anger | 0.19 | [0.12, 0.27] |
| Sadness | 0.25 | [0.17, 0.33] |
| Anxiety | 0.29 | [0.21, 0.39] |
| Secondary Appraisals |  |  |
| Liking | 0.11 | [0.06, 0.18] |
| Beauty | 0.14 | [0.08, 0.22] |
| Goodness | 0.21 | [0.13, 0.29] |
| Meaning | 0.15 | [0.08, 0.24] |
| Complex | 0.16 | [0.08, 0.25] |
| Interest | 0.15 | [0.09, 0.23] |
| Strength | 0.17 | [0.11, 0.25] |
| Activity | 0.08 | [0.03, 0.15] |
| Cognitive to visceral | 0.11 | [0.06, 0.17] |
| Pay to see again / in person | 0.37 | [0.28, 0.46] |
| Feel better | 0.08 | [0.03, 0.14] |
| Better person | 0.17 | [0.11, 0.24] |
| Change you | 0.32 | [0.25, 0.41] |

**Supplementary Table S3.** Descriptive statistics of participant ratings of secondary appraisals, including aesthetic judgements, implications, and impact measures, by condition (overall & by-artwork)

**S3A.** Summary statistics of aesthetic judgement ratings.

*Note.* All variables were rated on 7pt. bipolar scales. Higher values indicate agreement with the name of the variable (i.e., for ‘Liking’: 1 = ‘Something I disliked’, 7 = ‘Something I personally liked’).

| Condition | Artwork | *n* | Liking | | Beauty | | Goodness | | Meaning | | Complex | | Interest | | Strength | | Activity | |
| --- | --- | --- | --- | --- | --- | --- | --- | --- | --- | --- | --- | --- | --- | --- | --- | --- | --- | --- |
|  |  |  | *M* | *SD* | *M* | *SD* | *M* | *SD* | *M* | *SD* | *M* | *SD* | *M* | *SD* | *M* | *SD* | *M* | *SD* |
| Museum | Total | 745 | 4.84 | 1.79 | 5.07 | 1.57 | 5.34 | 1.57 | 4.90 | 1.66 | 4.92 | 1.70 | 5.30 | 1.64 | 5.06 | 1.58 | 4.55 | 1.78 |
| Digital | Total | 904 | 4.29 | 1.84 | 4.55 | 1.65 | 4.88 | 1.65 | 4.40 | 1.71 | 4.40 | 1.70 | 4.66 | 1.80 | 4.60 | 1.58 | 4.32 | 1.66 |
| Museum | Basquiat | 87 | 4.92 | 1.77 | 4.75 | 1.50 | 5.22 | 1.50 | 5.80 | 1.46 | 5.69 | 1.45 | 5.93 | 1.35 | 5.89 | 1.38 | 5.82 | 1.40 |
|  | Katz | 100 | 4.86 | 1.57 | 5.28 | 1.30 | 5.36 | 1.30 | 4.60 | 1.71 | 4.17 | 1.79 | 4.86 | 1.61 | 4.70 | 1.64 | 3.86 | 1.74 |
|  | Kupka | 90 | 4.18 | 1.85 | 4.87 | 1.56 | 4.88 | 1.56 | 4.44 | 1.66 | 4.61 | 1.71 | 4.84 | 1.74 | 4.61 | 1.50 | 3.90 | 1.75 |
|  | Magritte | 111 | 4.50 | 1.94 | 4.86 | 1.71 | 5.00 | 1.71 | 4.70 | 1.67 | 5.10 | 1.54 | 5.26 | 1.74 | 4.80 | 1.62 | 4.46 | 1.80 |
|  | Miro | 100 | 4.82 | 1.83 | 4.76 | 1.62 | 5.40 | 1.62 | 4.76 | 1.67 | 4.80 | 1.83 | 5.36 | 1.74 | 4.92 | 1.63 | 5.08 | 1.68 |
|  | Monet | 97 | 5.55 | 1.57 | 5.78 | 1.34 | 5.95 | 1.34 | 5.08 | 1.58 | 4.81 | 1.68 | 5.57 | 1.49 | 5.19 | 1.46 | 4.38 | 1.65 |
|  | Rothko | 79 | 4.81 | 1.82 | 4.78 | 1.66 | 5.21 | 1.66 | 4.53 | 1.57 | 4.97 | 1.56 | 5.13 | 1.57 | 5.00 | 1.44 | 4.40 | 1.73 |
|  | Troger | 81 | 5.11 | 1.69 | 5.44 | 1.55 | 5.73 | 1.55 | 5.42 | 1.53 | 5.35 | 1.57 | 5.48 | 1.57 | 5.52 | 1.57 | 4.56 | 1.64 |
| Digital | Basquiat | 113 | 3.81 | 1.98 | 3.52 | 1.61 | 4.50 | 1.61 | 4.66 | 1.65 | 5.19 | 1.57 | 4.89 | 1.79 | 4.90 | 1.67 | 5.24 | 1.51 |
|  | Katz | 113 | 4.26 | 1.62 | 4.73 | 1.47 | 4.92 | 1.47 | 4.15 | 1.44 | 3.57 | 1.35 | 4.29 | 1.63 | 4.16 | 1.40 | 3.78 | 1.43 |
|  | Kupka | 113 | 4.41 | 1.71 | 4.73 | 1.51 | 4.67 | 1.51 | 4.00 | 1.59 | 4.09 | 1.62 | 4.54 | 1.78 | 4.36 | 1.55 | 3.83 | 1.64 |
|  | Magritte | 113 | 4.96 | 1.68 | 4.86 | 1.45 | 5.49 | 1.45 | 5.44 | 1.55 | 5.62 | 1.32 | 5.82 | 1.28 | 5.32 | 1.40 | 5.29 | 1.42 |
|  | Miro | 113 | 4.31 | 1.88 | 4.19 | 1.75 | 4.64 | 1.75 | 4.08 | 1.46 | 3.76 | 1.46 | 4.65 | 1.70 | 4.43 | 1.39 | 4.75 | 1.28 |
|  | Monet | 113 | 5.10 | 1.61 | 5.42 | 1.50 | 5.49 | 1.50 | 4.44 | 1.68 | 4.35 | 1.49 | 4.72 | 1.70 | 4.67 | 1.43 | 3.63 | 1.49 |
|  | Rothko | 113 | 3.12 | 1.69 | 3.81 | 1.60 | 3.81 | 1.60 | 2.99 | 1.52 | 3.27 | 1.62 | 3.36 | 1.84 | 3.59 | 1.68 | 3.20 | 1.62 |
|  | Troger | 113 | 4.34 | 1.84 | 5.15 | 1.36 | 5.50 | 1.36 | 5.42 | 1.44 | 5.36 | 1.49 | 5.04 | 1.69 | 5.32 | 1.33 | 4.82 | 1.48 |

**S3B.** Summary statistics of implications and impact ratings.

*Note.* ‘Cognitive to Visceral’ was rated on a 7 pt. bipolar scale (1=’More detached and cognitive’, 7=’Very emotional and visceral’; item was reverse scored). ‘Pay to see again/in person’ was rated on a 7 pt. scale (1=‘Not at all’, 7=‘Very much so’). Remaining items were rated on 11pt bipolar scales: Feel better (0=‘worse’ to 10='better’); Better person (0=‘worse person’ to 10=‘better person’); Change you (0=‘Not at all’ to 10=‘Very much so’).

| Condition | Artwork | *n* | Cognitive to Visceral | | Pay to see again / in person | | Feel better | | Better person | | Change you | |
| --- | --- | --- | --- | --- | --- | --- | --- | --- | --- | --- | --- | --- |
|  |  |  | *M* | *SD* | *M* | *SD* | *M* | *SD* | *M* | *SD* | *M* | *SD* |
| Museum | Total | 745 | 3.67 | 1.75 | 3.60 | 1.88 | 5.99 | 1.98 | 5.82 | 1.59 | 3.87 | 2.71 |
| Digital | Total | 904 | 3.75 | 1.72 | 3.24 | 1.99 | 5.52 | 2.08 | 5.31 | 1.23 | 3.18 | 2.54 |
| Museum | Basquiat | 87 | 4.12 | 1.80 | 4.09 | 1.94 | 5.36 | 1.72 | 5.76 | 1.53 | 4.14 | 2.47 |
|  | Katz | 100 | 3.43 | 1.71 | 2.91 | 1.67 | 5.93 | 1.87 | 5.68 | 1.55 | 3.73 | 2.70 |
|  | Kupka | 90 | 3.26 | 1.81 | 2.81 | 1.66 | 5.85 | 1.93 | 5.97 | 1.57 | 3.77 | 2.96 |
|  | Magritte | 111 | 3.17 | 1.68 | 3.26 | 1.80 | 5.90 | 2.20 | 5.81 | 1.74 | 3.91 | 2.84 |
|  | Miro | 100 | 3.43 | 1.58 | 3.86 | 1.82 | 6.05 | 1.75 | 5.67 | 1.60 | 3.68 | 2.74 |
|  | Monet | 97 | 4.14 | 1.73 | 4.39 | 1.84 | 6.73 | 1.90 | 5.88 | 1.41 | 3.98 | 2.59 |
|  | Rothko | 79 | 3.43 | 1.53 | 3.68 | 1.93 | 5.96 | 2.07 | 5.74 | 1.63 | 3.77 | 2.59 |
|  | Troger | 81 | 4.64 | 1.69 | 3.91 | 1.84 | 6.08 | 2.18 | 6.09 | 1.71 | 4.03 | 2.80 |
| Digital | Basquiat | 113 | 3.82 | 1.69 | 2.98 | 1.95 | 4.73 | 2.01 | 4.96 | 1.30 | 3.19 | 2.41 |
|  | Katz | 113 | 3.82 | 1.58 | 2.85 | 1.74 | 5.66 | 1.84 | 5.30 | 1.11 | 3.07 | 2.47 |
|  | Kupka | 113 | 3.30 | 1.48 | 3.09 | 1.82 | 5.80 | 1.83 | 5.19 | 1.08 | 2.87 | 2.46 |
|  | Magritte | 113 | 3.68 | 1.79 | 4.04 | 2.15 | 6.04 | 1.97 | 5.54 | 1.29 | 4.02 | 2.70 |
|  | Miro | 113 | 3.91 | 1.62 | 3.20 | 1.96 | 6.02 | 2.17 | 5.47 | 1.30 | 3.42 | 2.50 |
|  | Monet | 113 | 4.17 | 1.58 | 3.78 | 2.03 | 6.38 | 2.07 | 5.63 | 1.32 | 3.32 | 2.60 |
|  | Rothko | 113 | 3.12 | 1.85 | 2.35 | 1.72 | 4.50 | 1.91 | 5.03 | 1.02 | 2.22 | 2.43 |
|  | Troger | 113 | 4.16 | 1.90 | 3.65 | 2.06 | 5.01 | 2.06 | 5.37 | 1.24 | 3.36 | 2.46 |

**Supplementary Table S4.** Two-way ANOVA results considering judgement ratings across levels of condition and artwork.

*Note.* Items ‘Liking’ to ‘Pay to see again/in person’ were rated on 7 pt. scales (1-7). Final items, ‘Feel better,’ ‘Better person,’ ‘Change you’ were rated on 11 pt. scales (0-10). Significance values were interpreted via Bonferroni correction (p = 0.05 / 13 analyses = 0.0038).

| Variable | Term | *df* | Sum Sq. | Mean Sq. | Statistic | *p* | η^2^ |
| --- | --- | --- | --- | --- | --- | --- | --- |
| Liking | Condition | 1 | 123.26 | 123.26 | 39.88 | < .001*** | 0.02 |
|  | Artwork | 7 | 249.21 | 35.60 | 11.52 | < .001*** | 0.04 |
|  | Condition × Artwork | 7 | 164.02 | 23.43 | 7.58 | < .001*** | 0.03 |
|  | Residuals | 1,620 | 5,006.79 | 3.09 |  |  | 0.90 |
| Beauty | Condition | 1 | 108.71 | 108.71 | 46.08 | < .001*** | 0.02 |
|  | Artwork | 7 | 380.80 | 54.40 | 23.06 | < .001*** | 0.09 |
|  | Condition × Artwork | 7 | 60.24 | 8.61 | 3.65 | .001*** | 0.01 |
|  | Residuals | 1,630 | 3,845.25 | 2.36 |  |  | 0.87 |
| Goodness | Condition | 1 | 87.26 | 87.26 | 39.60 | < .001*** | 0.02 |
|  | Artwork | 7 | 264.76 | 37.82 | 17.17 | < .001*** | 0.07 |
|  | Condition × Artwork | 7 | 104.27 | 14.90 | 6.76 | < .001*** | 0.03 |
|  | Residuals | 1,628 | 3,586.95 | 2.20 |  |  | 0.89 |
| Meaning | Condition | 1 | 104.37 | 104.37 | 42.01 | < .001*** | 0.02 |
|  | Artwork | 7 | 478.69 | 68.38 | 27.52 | < .001*** | 0.10 |
|  | Condition × Artwork | 7 | 170.54 | 24.36 | 9.81 | < .001*** | 0.04 |
|  | Residuals | 1,630 | 4,049.62 | 2.48 |  |  | 0.84 |
| Complex | Condition | 1 | 109.50 | 109.50 | 44.63 | < .001*** | 0.02 |
|  | Artwork | 7 | 608.76 | 86.97 | 35.45 | < .001*** | 0.13 |
|  | Condition × Artwork | 7 | 153.95 | 21.99 | 8.96 | < .001*** | 0.03 |
|  | Residuals | 1,629 | 3,996.68 | 2.45 |  |  | 0.82 |
| Interest | Condition | 1 | 163.91 | 163.91 | 59.95 | < .001*** | 0.03 |
|  | Artwork | 7 | 311.67 | 44.52 | 16.29 | < .001*** | 0.06 |
|  | Condition × Artwork | 7 | 156.55 | 22.36 | 8.18 | < .001*** | 0.03 |
|  | Residuals | 1,631 | 4,458.90 | 2.73 |  |  | 0.88 |
| Strength | Condition | 1 | 86.78 | 86.78 | 38.06 | < .001*** | 0.02 |
|  | Artwork | 7 | 278.12 | 39.73 | 17.42 | < .001*** | 0.07 |
|  | Condition × Artwork | 7 | 114.23 | 16.32 | 7.16 | < .001*** | 0.03 |
|  | Residuals | 1,630 | 3,717.09 | 2.28 |  |  | 0.89 |
| Activity | Condition | 1 | 21.55 | 21.55 | 8.66 | .003** | 0.00 |
|  | Artwork | 7 | 624.96 | 89.28 | 35.87 | < .001*** | 0.13 |
|  | Condition × Artwork | 7 | 140.15 | 20.02 | 8.04 | < .001*** | 0.03 |
|  | Residuals | 1,630 | 4,056.88 | 2.49 |  |  | 0.84 |
| Cognitive to Visceral | Condition | 1 | 2.27 | 2.27 | 0.79 | .373 | 0.00 |
|  | Artwork | 7 | 228.78 | 32.68 | 11.40 | < .001*** | 0.05 |
|  | Condition × Artwork | 7 | 51.77 | 7.40 | 2.58 | .012* | 0.01 |
|  | Residuals | 1,615 | 4,628.04 | 2.87 |  |  | 0.94 |
| Pay to see again / in person | Condition | 1 | 50.27 | 50.27 | 14.23 | < .001*** | 0.01 |
|  | Artwork | 7 | 276.28 | 39.47 | 11.18 | < .001*** | 0.04 |
|  | Condition × Artwork | 7 | 176.09 | 25.16 | 7.12 | < .001*** | 0.03 |
|  | Residuals | 1,620 | 5,720.71 | 3.53 |  |  | 0.92 |
| Feel better | Condition | 1 | 90.49 | 90.49 | 23.20 | < .001*** | 0.01 |
|  | Artwork | 7 | 356.36 | 50.91 | 13.05 | < .001*** | 0.05 |
|  | Condition × Artwork | 7 | 104.90 | 14.99 | 3.84 | < .001*** | 0.02 |
|  | Residuals | 1,617 | 6,308.44 | 3.90 |  |  | 0.92 |
| Better person | Condition | 1 | 103.25 | 103.25 | 52.92 | < .001*** | 0.03 |
|  | Artwork | 7 | 33.73 | 4.82 | 2.47 | .016* | 0.01 |
|  | Condition × Artwork | 7 | 23.80 | 3.40 | 1.74 | .095 | 0.01 |
|  | Residuals | 1,615 | 3,150.73 | 1.95 |  |  | 0.95 |
| Change you | Condition | 1 | 189.74 | 189.74 | 27.99 | < .001*** | 0.02 |
|  | Artwork | 7 | 140.26 | 20.04 | 2.96 | .004** | 0.01 |
|  | Condition × Artwork | 7 | 84.64 | 12.09 | 1.78 | .087 | 0.01 |
|  | Residuals | 1,616 | 10,955.20 | 6.78 |  |  | 0.96 |

**Supplementary Table S5.** Descriptive statistics of phenomenal state ratings, by condition (overall & by-artwork)

*Note.* All items, reported in Table S5A-B, were rated on an 11 pt. scale (0=‘Not at all’, 10=‘Very much so’), in response to the prompt ‘While viewing the artwork, I felt…’

**S5A.** Ratings of first eight of sixteen phenomenal states.

| Condition | Artwork | *n* | Love | | Happiness | | Playfulness | | Pleasure | | Sense of beauty | | Insight | | Moved | | Transformation | |
| --- | --- | --- | --- | --- | --- | --- | --- | --- | --- | --- | --- | --- | --- | --- | --- | --- | --- | --- |
|  |  |  | *M* | *SD* | *M* | *SD* | *M* | *SD* | *M* | *SD* | *M* | *SD* | *M* | *SD* | *M* | *SD* | *M* | *SD* |
| Museum | Total | 745 | 2.86 | 3.11 | 3.70 | 3.11 | 2.92 | 1.57 | 4.55 | 3.09 | 5.09 | 3.22 | 3.16 | 2.96 | 3.88 | 3.01 | 2.57 | 2.79 |
| Digital | Total | 904 | 2.34 | 2.75 | 3.25 | 2.99 | 3.53 | 1.65 | 3.92 | 3.12 | 4.64 | 3.08 | 2.47 | 2.78 | 3.45 | 2.82 | 2.32 | 2.66 |
| Museum | Basquiat | 87 | 2.20 | 2.98 | 2.26 | 2.93 | 2.52 | 1.50 | 3.36 | 3.02 | 3.69 | 3.01 | 4.01 | 3.05 | 5.10 | 2.77 | 2.91 | 2.67 |
|  | Katz | 100 | 2.60 | 2.99 | 3.12 | 3.09 | 1.79 | 1.30 | 4.17 | 3.01 | 5.24 | 3.15 | 2.44 | 2.93 | 3.65 | 3.15 | 1.51 | 2.26 |
|  | Kupka | 90 | 2.20 | 2.74 | 3.50 | 2.79 | 2.99 | 1.56 | 4.31 | 2.79 | 4.42 | 3.01 | 3.14 | 2.69 | 2.62 | 2.50 | 3.26 | 2.99 |
|  | Magritte | 111 | 2.52 | 3.06 | 3.47 | 3.03 | 3.32 | 1.71 | 4.22 | 3.23 | 4.03 | 3.28 | 3.53 | 3.21 | 2.86 | 2.93 | 2.78 | 2.89 |
|  | Miro | 100 | 2.50 | 3.02 | 4.00 | 3.03 | 4.53 | 1.62 | 4.71 | 3.15 | 4.61 | 3.13 | 3.03 | 3.06 | 3.53 | 2.82 | 2.23 | 2.86 |
|  | Monet | 97 | 4.34 | 3.19 | 5.66 | 2.50 | 3.05 | 1.34 | 6.39 | 2.68 | 7.57 | 1.89 | 2.85 | 2.75 | 4.64 | 2.77 | 2.35 | 2.56 |
|  | Rothko | 79 | 2.95 | 3.07 | 4.48 | 3.11 | 3.29 | 1.66 | 4.62 | 2.93 | 4.73 | 3.35 | 2.78 | 2.82 | 3.90 | 3.06 | 3.00 | 3.08 |
|  | Troger | 81 | 3.69 | 3.29 | 3.02 | 3.26 | 1.67 | 1.55 | 4.52 | 3.11 | 6.58 | 2.82 | 3.54 | 2.89 | 5.19 | 3.02 | 2.69 | 2.69 |
| Digital | Basquiat | 113 | 1.19 | 1.92 | 2.02 | 2.57 | 3.30 | 1.61 | 2.81 | 2.94 | 2.97 | 2.88 | 2.53 | 2.75 | 3.51 | 2.72 | 2.42 | 2.57 |
|  | Katz | 113 | 2.81 | 2.73 | 3.50 | 2.88 | 2.71 | 1.47 | 3.91 | 2.85 | 5.05 | 2.76 | 2.15 | 2.67 | 2.96 | 2.71 | 1.68 | 2.35 |
|  | Kupka | 113 | 1.94 | 2.49 | 3.48 | 2.71 | 3.76 | 1.51 | 4.27 | 2.92 | 5.07 | 3.05 | 2.51 | 2.85 | 3.05 | 2.71 | 2.45 | 2.66 |
|  | Magritte | 113 | 2.12 | 2.76 | 3.85 | 3.16 | 5.09 | 1.45 | 4.86 | 3.15 | 4.98 | 3.01 | 3.94 | 3.20 | 4.53 | 2.85 | 3.57 | 3.09 |
|  | Miro | 113 | 2.85 | 3.03 | 4.31 | 3.24 | 6.28 | 1.75 | 5.22 | 3.08 | 4.14 | 3.01 | 2.35 | 2.53 | 3.28 | 2.76 | 2.41 | 2.73 |
|  | Monet | 113 | 3.84 | 2.94 | 4.88 | 2.77 | 3.31 | 1.50 | 5.58 | 2.83 | 6.57 | 2.65 | 2.13 | 2.45 | 4.05 | 2.82 | 2.30 | 2.65 |
|  | Rothko | 113 | 1.54 | 2.18 | 2.35 | 2.57 | 2.64 | 1.60 | 2.52 | 2.51 | 3.10 | 2.59 | 1.80 | 2.51 | 2.00 | 2.08 | 1.58 | 2.20 |
|  | Troger | 113 | 2.45 | 2.88 | 1.63 | 2.42 | 1.12 | 1.36 | 2.19 | 2.80 | 5.27 | 3.08 | 2.37 | 2.75 | 4.17 | 3.05 | 2.18 | 2.59 |

**S5B.** Ratings of second eight of sixteen phenomenal states.

| Condition | Artwork | *n* | Change in myself | | Thrilled | | Active engagement | | Boredom | | Shock | | Anger | | Sadness | | Anxiety | |
| --- | --- | --- | --- | --- | --- | --- | --- | --- | --- | --- | --- | --- | --- | --- | --- | --- | --- | --- |
|  |  |  | *M* | *SD* | *M* | *SD* | *M* | *SD* | *M* | *SD* | *M* | *SD* | *M* | *SD* | *M* | *SD* | *M* | *SD* |
| Museum | Total | 745 | 1.89 | 2.45 | 2.47 | 2.80 | 4.45 | 3.15 | 1.92 | 2.52 | 1.72 | 2.46 | 1.35 | 2.29 | 3.00 | 3.18 | 2.02 | 2.75 |
| Digital | Total | 904 | 2.24 | 2.54 | 3.08 | 2.77 | 4.88 | 3.10 | 2.06 | 2.61 | 1.42 | 2.28 | 1.04 | 1.91 | 2.10 | 2.60 | 1.61 | 2.39 |
| Museum | Basquiat | 87 | 2.29 | 2.43 | 3.10 | 3.00 | 5.57 | 2.95 | 1.23 | 1.92 | 3.64 | 2.99 | 4.02 | 3.03 | 4.49 | 3.24 | 3.69 | 3.19 |
|  | Katz | 100 | 1.46 | 2.28 | 1.52 | 2.37 | 4.07 | 3.36 | 1.97 | 2.51 | 0.70 | 1.80 | 0.52 | 1.22 | 3.70 | 3.34 | 1.40 | 2.53 |
|  | Kupka | 90 | 1.92 | 2.22 | 2.30 | 2.47 | 4.53 | 2.86 | 2.61 | 2.70 | 1.38 | 1.99 | 1.18 | 2.08 | 1.79 | 2.71 | 2.11 | 2.94 |
|  | Magritte | 111 | 1.99 | 2.49 | 2.67 | 2.86 | 4.10 | 3.31 | 2.28 | 2.59 | 1.99 | 2.48 | 1.43 | 2.26 | 2.77 | 3.04 | 2.56 | 3.02 |
|  | Miro | 100 | 1.73 | 2.45 | 2.65 | 2.85 | 4.80 | 3.19 | 1.74 | 2.36 | 1.76 | 2.28 | 1.11 | 1.95 | 2.34 | 2.80 | 1.70 | 2.29 |
|  | Monet | 97 | 1.75 | 2.48 | 2.42 | 2.67 | 3.66 | 3.08 | 1.52 | 2.40 | 0.47 | 1.17 | 0.52 | 1.59 | 1.80 | 2.45 | 0.71 | 1.49 |
|  | Rothko | 79 | 1.75 | 2.45 | 2.77 | 3.15 | 4.59 | 3.16 | 2.48 | 2.97 | 1.85 | 2.60 | 1.25 | 2.26 | 2.28 | 2.77 | 2.14 | 2.89 |
|  | Troger | 81 | 2.36 | 2.79 | 2.42 | 2.88 | 4.47 | 2.90 | 1.53 | 2.41 | 2.25 | 2.75 | 0.96 | 1.68 | 5.10 | 3.44 | 1.96 | 2.45 |
| Digital | Basquiat | 113 | 2.18 | 2.33 | 4.10 | 2.76 | 5.14 | 2.96 | 1.30 | 1.84 | 2.98 | 3.09 | 2.52 | 2.69 | 2.28 | 2.62 | 3.11 | 3.04 |
|  | Katz | 113 | 1.95 | 2.28 | 2.09 | 2.31 | 4.35 | 2.77 | 2.58 | 2.71 | 0.46 | 0.90 | 0.44 | 1.09 | 2.33 | 2.77 | 0.88 | 1.80 |
|  | Kupka | 113 | 2.19 | 2.57 | 2.82 | 2.53 | 4.96 | 3.05 | 2.09 | 2.71 | 0.88 | 1.68 | 0.69 | 1.47 | 1.35 | 1.97 | 1.21 | 1.87 |
|  | Magritte | 113 | 3.37 | 2.94 | 4.33 | 3.16 | 6.33 | 3.05 | 0.93 | 1.65 | 2.07 | 2.51 | 0.98 | 1.89 | 1.93 | 2.45 | 1.80 | 2.56 |
|  | Miro | 113 | 2.42 | 2.73 | 3.26 | 2.71 | 5.16 | 3.06 | 1.88 | 2.58 | 1.10 | 1.99 | 0.73 | 1.62 | 1.12 | 1.88 | 1.12 | 1.86 |
|  | Monet | 113 | 2.33 | 2.48 | 2.50 | 2.46 | 4.54 | 2.97 | 2.03 | 2.58 | 0.34 | 1.06 | 0.34 | 0.91 | 2.25 | 2.80 | 0.81 | 1.63 |
|  | Rothko | 113 | 1.35 | 1.93 | 2.13 | 2.53 | 3.35 | 2.98 | 3.45 | 2.93 | 1.31 | 2.31 | 1.19 | 2.06 | 1.55 | 2.09 | 1.37 | 2.37 |
|  | Troger | 113 | 2.17 | 2.57 | 3.45 | 2.81 | 5.19 | 3.16 | 2.23 | 2.83 | 2.19 | 2.47 | 1.42 | 2.05 | 3.96 | 3.00 | 2.56 | 2.65 |

**Supplementary Table S6.** Two-way ANOVA results of overall magnitude of phenomenal state ratings.

*Note.* Overall magnitude refers to ratings averaged across all 16 phenomenal states (each rated on 11 pt. scale, 0-10; see Supplementary Table S5 for summary statistics).

**S6A.** Two-way ANOVA results table.

|  | Df | Sum Sq | Mean Sq | F value | Pr(>F) |
| --- | --- | --- | --- | --- | --- |
| Condition | 1 | 261 | 261.17 | 29.16 | < 0.001 *** |
| Artwork | 7 | 1567 | 223.84 | 24.99 | < 0.001 *** |
| Condition : Artwork | 7 | 1181 | 168.76 | 18.84 | < 0.001 *** |
| Residuals | 26361 | 236101 | 8.96 |  |  |

**S6B.** Pairwise contrast, Museum – Digital, averaged over levels of artwork.

| Contrast | estimate | SE | df | t.ratio | p.value | effect.size |
| --- | --- | --- | --- | --- | --- | --- |
| Museum - Digital | 0.214 | 0.037 | 26361 | 5.762 | <.0001 *** | 0.072 |

**S6C.** Pairwise contrasts, Museum – Digital, within each level of artwork.

| Artwork | estimate | SE | df | t.ratio | p.value | effect.size |
| --- | --- | --- | --- | --- | --- | --- |
| Basquiat | 0.608 | 0.107 | 26361 | 5.698 | <.0001 *** | 0.203 |
| Katz | -0.0007 | 0.103 | 26361 | -0.007 | 0.995 | -0.0002 |
| Kupka | 0.096 | 0.106 | 26361 | 0.906 | 0.365 | 0.032 |
| Magritte | -0.509 | 0.100 | 26361 | -5.095 | <.0001 *** | -0.170 |
| Miro | -0.041 | 0.103 | 26361 | -0.395 | 0.693 | -0.014 |
| Monet | 0.118 | 0.104 | 26361 | 1.137 | 0.256 | 0.039 |
| Rothko | 0.978 | 0.110 | 26361 | 8.911 | <.0001 *** | 0.327 |
| Troger | 0.464 | 0.109 | 26361 | 4.257 | <.0001 *** | 0.155 |

**Supplementary Table S7.** Two-way ANOVA results considering individual phenomenal state ratings across levels of condition and artwork.

*Note.* All items were rated on 11 pt. scales (0-10). Significance values were interpreted via Bonferroni correction (p = 0.05 / 16 analyses = 0.0031).

| Variable | Term | *df* | Sum Sq. | Mean Sq. | Statistic | *p* | η^2^ |
| --- | --- | --- | --- | --- | --- | --- | --- |
| Love | Condition | 1 | 110.41 | 110.41 | 13.79 | < .001*** | 0.01 |
|  | Artwork | 7 | 790.90 | 112.99 | 14.11 | < .001*** | 0.06 |
|  | Condition × Artwork | 7 | 145.72 | 20.82 | 2.60 | .011* | 0.01 |
|  | Residuals | 1,633 | 13,077.52 | 8.01 |  |  | 0.93 |
| Happiness | Condition | 1 | 81.35 | 81.35 | 9.81 | .002** | 0.01 |
|  | Artwork | 7 | 1,428.53 | 204.08 | 24.60 | < .001*** | 0.09 |
|  | Condition × Artwork | 7 | 296.27 | 42.32 | 5.10 | < .001*** | 0.02 |
|  | Residuals | 1,632 | 13,536.15 | 8.29 |  |  | 0.88 |
| Playfulness | Condition | 1 | 147.33 | 147.33 | 17.25 | < .001*** | 0.01 |
|  | Artwork | 7 | 2,236.90 | 319.56 | 37.41 | < .001*** | 0.13 |
|  | Condition × Artwork | 7 | 295.56 | 42.22 | 4.94 | < .001*** | 0.02 |
|  | Residuals | 1,633 | 13,950.75 | 8.54 |  |  | 0.84 |
| Pleasure | Condition | 1 | 160.03 | 160.03 | 18.52 | < .001*** | 0.01 |
|  | Artwork | 7 | 1,379.36 | 197.05 | 22.80 | < .001*** | 0.09 |
|  | Condition × Artwork | 7 | 422.05 | 60.29 | 6.98 | < .001*** | 0.03 |
|  | Residuals | 1,633 | 14,113.51 | 8.64 |  |  | 0.88 |
| Sense of beauty | Condition | 1 | 81.29 | 81.29 | 9.46 | .002** | 0.00 |
|  | Artwork | 7 | 1,979.72 | 282.82 | 32.91 | < .001*** | 0.12 |
|  | Condition × Artwork | 7 | 294.62 | 42.09 | 4.90 | < .001*** | 0.02 |
|  | Residuals | 1,633 | 14,031.94 | 8.59 |  |  | 0.86 |
| Insight | Condition | 1 | 192.98 | 192.98 | 24.23 | < .001*** | 0.01 |
|  | Artwork | 7 | 365.62 | 52.23 | 6.56 | < .001*** | 0.03 |
|  | Condition × Artwork | 7 | 121.62 | 17.37 | 2.18 | .033* | 0.01 |
|  | Residuals | 1,633 | 13,004.06 | 7.96 |  |  | 0.95 |
| Moved | Condition | 1 | 77.44 | 77.44 | 9.89 | .002** | 0.01 |
|  | Artwork | 7 | 642.18 | 91.74 | 11.72 | < .001*** | 0.05 |
|  | Condition × Artwork | 7 | 474.30 | 67.76 | 8.66 | < .001*** | 0.03 |
|  | Residuals | 1,631 | 12,766.44 | 7.83 |  |  | 0.91 |
| Transformation | Condition | 1 | 24.74 | 24.74 | 3.45 | .064 | 0.00 |
|  | Artwork | 7 | 322.97 | 46.14 | 6.43 | < .001*** | 0.03 |
|  | Condition × Artwork | 7 | 165.49 | 23.64 | 3.29 | .002** | 0.01 |
|  | Residuals | 1,629 | 11,692.89 | 7.18 |  |  | 0.96 |
| Change in myself | Condition | 1 | 49.86 | 49.86 | 8.14 | .004** | 0.00 |
|  | Artwork | 7 | 186.88 | 26.70 | 4.36 | < .001*** | 0.02 |
|  | Condition × Artwork | 7 | 119.17 | 17.02 | 2.78 | .007** | 0.01 |
|  | Residuals | 1,633 | 10,005.04 | 6.13 |  |  | 0.97 |
| Thrilled | Condition | 1 | 154.11 | 154.11 | 20.83 | < .001*** | 0.01 |
|  | Artwork | 7 | 547.84 | 78.26 | 10.58 | < .001*** | 0.04 |
|  | Condition × Artwork | 7 | 165.51 | 23.64 | 3.20 | .002** | 0.01 |
|  | Residuals | 1,633 | 12,079.83 | 7.40 |  |  | 0.93 |
| Sense of active engagement | Condition | 1 | 75.04 | 75.04 | 8.04 | .005** | 0.00 |
|  | Artwork | 7 | 422.17 | 60.31 | 6.46 | < .001*** | 0.03 |
|  | Condition × Artwork | 7 | 363.28 | 51.90 | 5.56 | < .001*** | 0.02 |
|  | Residuals | 1,633 | 15,241.53 | 9.33 |  |  | 0.95 |
| Boredom | Condition | 1 | 7.70 | 7.70 | 1.23 | .268 | 0.00 |
|  | Artwork | 7 | 409.74 | 58.53 | 9.31 | < .001*** | 0.04 |
|  | Condition × Artwork | 7 | 212.20 | 30.31 | 4.82 | < .001*** | 0.02 |
|  | Residuals | 1,633 | 10,264.36 | 6.29 |  |  | 0.94 |
| Shock | Condition | 1 | 37.69 | 37.69 | 7.76 | .005** | 0.00 |
|  | Artwork | 7 | 1,262.93 | 180.42 | 37.14 | < .001*** | 0.14 |
|  | Condition × Artwork | 7 | 30.01 | 4.29 | 0.88 | .519 | 0.00 |
|  | Residuals | 1,633 | 7,932.77 | 4.86 |  |  | 0.86 |
| Anger | Condition | 1 | 39.04 | 39.04 | 10.55 | .001** | 0.01 |
|  | Artwork | 7 | 1,061.64 | 151.66 | 40.98 | < .001*** | 0.15 |
|  | Condition × Artwork | 7 | 106.87 | 15.27 | 4.13 | < .001*** | 0.01 |
|  | Residuals | 1,633 | 6,043.31 | 3.70 |  |  | 0.83 |
| Sadness | Condition | 1 | 331.44 | 331.44 | 44.79 | < .001*** | 0.02 |
|  | Artwork | 7 | 1,361.88 | 194.55 | 26.29 | < .001*** | 0.10 |
|  | Condition × Artwork | 7 | 210.14 | 30.02 | 4.06 | < .001*** | 0.02 |
|  | Residuals | 1,633 | 12,084.79 | 7.40 |  |  | 0.86 |
| Anxiety | Condition | 1 | 68.26 | 68.26 | 11.41 | .001*** | 0.01 |
|  | Artwork | 7 | 943.30 | 134.76 | 22.53 | < .001*** | 0.09 |
|  | Condition × Artwork | 7 | 89.63 | 12.80 | 2.14 | .037* | 0.01 |
|  | Residuals | 1,633 | 9,766.47 | 5.98 |  |  | 0.90 |

**Supplementary Table S8.** Results of auxiliary analyses assessing differences between experience type groups within the Digital Cohort.

*Note*. General art liking and knowledge, as well as the three impact measures (‘Feel better,’ ‘Better person,’ and ‘Change you’) were rated on 11 pt. scales (0-10). All other variables were rated on 7 pt. scales (1-7). Stars (*) indicate significant group differences at p < 0.002 (Bonferroni corrected for 21 comparisons; 0.05/26 = 0.0024). Pairwise comparisons indicated are also significant at p < 0.002.

|  | *Latent Profiles* | | | | | | | | | | *Difference statistic* | *Significant group-wise comparisons* |
| --- | --- | --- | --- | --- | --- | --- | --- | --- | --- | --- | --- | --- |
|  | Harmonious | | Novel | | Transformative | | Disengaged | | Negative | |  |  |
| *n* = 904 | 253 | | 183 | | 70 | | 304 | | 94 | |  |  |
|  | 27.99% | | 20.24% | | 7.74% | | 33.63% | | 10.40% | |  |  |
|  | *M* | *SE* | *M* | *SE* | *M* | *SE* | *M* | *SE* | *M* | *SE* | X^2^ |  |
| Age | 28.149 | 0.594 | 29.218 | 0.672 | 27.664 | 0.902 | 28.764 | 0.524 | 25.888 | 0.574 | 18.784 * | Nov./Neg., Dis./Neg. |
| General liking | 7.994 | 0.122 | 8.387 | 0.114 | 7.924 | 0.188 | 7.155 | 0.140 | 7.325 | 0.223 | 55.278 * | Har./Dis., Nov./Dis., Nov./Neg., Trn./Dis. |
| General knowledge | 4.739 | 0.143 | 5.476 | 0.167 | 5.399 | 0.237 | 4.424 | 0.147 | 4.296 | 0.254 | 33.936 * | Har./Nov., Nov./Dis., Nov./Neg., Trn./Dis., Trn./Neg. |
| Museum/gallery visits per year | 5.393 | 0.393 | 7.068 | 0.499 | 5.563 | 0.553 | 5.623 | 0.477 | 3.579 | 0.405 | 31.116 * | Har./Neg., Nov./Neg., Dis./Neg. |
| Gender |  |  |  |  |  |  |  |  |  |  | 14.366 |  |
| % Female | 76.3% | 0.030 | 77.4% | 0.032 | 70.5% | 0.057 | 66.1% | 0.028 | 76.5% | 0.050 |  |  |
| % Male | 20.1% | 0.028 | 18.0% | 0.030 | 23.7% | 0.053 | 31.3% | 0.027 | 21.1% | 0.048 |  |  |
| % Nonbinary/Other | 3.6% | 0.012 | 4.6% | 0.016 | 5.8% | 0.031 | 2.6% | 0.009 | 2.4% | 0.019 |  |  |
| View Number |  |  |  |  |  |  |  |  |  |  | 39.259 |  |
| 1 | 21.7% | 0.028 | 7.4% | 0.021 | 4.3% | 0.025 | 10.4% | 0.018 | 11.7% | 0.036 |  |  |
| 2 | 13.6% | 0.024 | 11.9% | 0.025 | 18.6% | 0.049 | 10.8% | 0.019 | 11.5% | 0.038 |  |  |
| 3 | 12.4% | 0.022 | 14.6% | 0.027 | 11.5% | 0.04 | 11.9% | 0.019 | 11.4% | 0.036 |  |  |
| 4 | 12.4% | 0.023 | 12.4% | 0.026 | 8.3% | 0.035 | 12.4% | 0.019 | 16.4% | 0.041 |  |  |
| 5 | 9.8% | 0.02 | 13.1% | 0.026 | 15.7% | 0.044 | 13.5% | 0.02 | 12.6% | 0.038 |  |  |
| 6 | 10.0% | 0.02 | 16.3% | 0.028 | 15.6% | 0.045 | 12.7% | 0.02 | 8.8% | 0.033 |  |  |
| 7 | 11.8% | 0.022 | 12.4% | 0.026 | 9.8% | 0.037 | 13.9% | 0.02 | 12.3% | 0.037 |  |  |
| 8 | 8.3% | 0.02 | 12.1% | 0.025 | 16.1% | 0.046 | 14.4% | 0.021 | 15.3% | 0.041 |  |  |
| Viewing Time (s) | 22.035 | 1.237 | 23.302 | 1.645 | 21.553 | 2.496 | 28.275 | 1.774 | 23.863 | 2.538 | 8.845 |  |
| Disliked - Liked | 5.05 | 0.088 | 6.098 | 0.083 | 4.032 | 0.219 | 3.111 | 0.096 | 2.707 | 0.196 | 708.872 * | all but Dis./Neg. |
| Ugly - Beautiful | 5.211 | 0.087 | 5.889 | 0.094 | 4.156 | 0.192 | 3.723 | 0.091 | 3.164 | 0.182 | 400.235 * | all but Trn./Dis., Dis./Neg. |
| Bad - Good | 5.318 | 0.087 | 6.017 | 0.096 | 5.022 | 0.163 | 4.041 | 0.09 | 4.059 | 0.197 | 267.713 * | all but Har./Trn., Dis./Neg |
| Meaningless - Meaningful | 4.645 | 0.09 | 5.469 | 0.117 | 5.049 | 0.189 | 3.432 | 0.105 | 4.273 | 0.203 | 187.892 * | all but Har./Trns, Har./Neg., Nov./Trn., Trn./Neg |
| Simple - Complex | 4.401 | 0.106 | 4.97 | 0.134 | 4.904 | 0.189 | 3.93 | 0.108 | 4.434 | 0.202 | 45.227 * | Har./Nov., Nov./Dis., Trn./Dis. |
| Boring - Interesting | 5.217 | 0.088 | 5.842 | 0.112 | 4.929 | 0.202 | 3.569 | 0.113 | 4.222 | 0.206 | 235.759 * | all but Har./Trn., Trn./Neg., Dis./Neg. |
| Weak - Strong | 4.828 | 0.084 | 5.68 | 0.101 | 5.083 | 0.183 | 3.69 | 0.093 | 4.408 | 0.201 | 224.12 * | all but Har./Trns, Har./Neg., Nov./Trn., Trn./Neg |
| Passive - Active | 4.438 | 0.098 | 5.199 | 0.118 | 5.056 | 0.178 | 3.517 | 0.099 | 4.308 | 0.209 | 142.275 * | all but Har./Neg., Nov./Trn., Trn./Neg. |
| Cognitive - Visceral | 3.721 | 0.098 | 4.874 | 0.115 | 4.699 | 0.207 | 2.788 | 0.096 | 3.985 | 0.227 | 223.978 * | all but Har./Neg., Nov./Trn., Trn./Neg. |
| Pay to see again / in person | 3.569 | 0.121 | 5.119 | 0.137 | 3.806 | 0.237 | 1.984 | 0.089 | 2.382 | 0.204 | 422.573 * | all but Har./Trn., Dis./Neg |
| How immersive | 4.255 | 0.094 | 5.595 | 0.091 | 4.631 | 0.187 | 2.792 | 0.093 | 3.573 | 0.191 | 495.44 * | all but Har./Trn. |
| Feel better | 6.273 | 0.107 | 7.673 | 0.135 | 4.498 | 0.264 | 4.552 | 0.082 | 3.212 | 0.215 | 593.166 * | all but Trn./Dis. |
| Better person | 5.467 | 0.072 | 6.157 | 0.119 | 5.242 | 0.16 | 4.914 | 0.046 | 4.593 | 0.167 | 134.167 * | all but Har./Neg., Trn./Dis., Trn./Neg., Dis./Neg. |
| Change you in some way | 3.02 | 0.167 | 5.14 | 0.169 | 4.826 | 0.241 | 1.8 | 0.137 | 3.027 | 0.255 | 291.56 * | all but Har./Neg., Nov./Trn |

**Supplementary Table S9.** Multinomial regression results, assessing potential differences between original and age matched datasets in predicting ExpType group assignment.

*Note.* Model ran was: rmultinom(ExTProfile ~ dataset * setting * artwork, data = both_data). Reference level of ‘ExTProfile’ was ‘Disengaged.’ Reference level of ‘dataset’ was ‘datasetMatched’ (referring to the reduced, age matched dataset). Reference level of ‘setting’ (i.e., condition) was ‘Museum’. Outputs at the level of artwork are not reported here, but are available upon request.

| y.level | term | estimate | std.error | statistic | p.value | conf.low | conf.high |
| --- | --- | --- | --- | --- | --- | --- | --- |
| Harmonious | (Intercept) | -0.580 | 0.506 | -1.147 | 0.252 | -1.572 | 0.411 |
|  | datasetOriginal | -0.232 | 0.661 | -0.351 | 0.726 | -1.528 | 1.064 |
|  | settingdigilab | -0.479 | 0.652 | -0.734 | 0.463 | -1.756 | 0.799 |
|  | datasetOriginal:settingdigilab | 0.473 | 0.838 | 0.564 | 0.572 | -1.169 | 2.115 |
| Novel | (Intercept) | -0.312 | 0.466 | -0.670 | 0.503 | -1.226 | 0.602 |
|  | datasetOriginal | -0.375 | 0.619 | -0.606 | 0.545 | -1.589 | 0.839 |
|  | settingdigilab | -0.517 | 0.600 | -0.861 | 0.389 | -1.694 | 0.660 |
|  | datasetOriginal:settingdigilab | 0.382 | 0.789 | 0.484 | 0.628 | -1.165 | 1.929 |
| Transformative | (Intercept) | 0.385 | 0.393 | 0.980 | 0.327 | -0.385 | 1.155 |
|  | datasetOriginal | -0.228 | 0.508 | -0.449 | 0.653 | -1.223 | 0.767 |
|  | settingdigilab | -0.747 | 0.510 | -1.464 | 0.143 | -1.747 | 0.253 |
|  | datasetOriginal:settingdigilab | 0.004 | 0.668 | 0.005 | 0.996 | -1.305 | 1.312 |
| Negative | (Intercept) | 0.793 | 0.365 | 2.172 | 0.030* | 0.077 | 1.509 |
|  | datasetOriginal | -0.250 | 0.470 | -0.530 | 0.596 | -1.172 | 0.673 |
|  | settingdigilab | -1.157 | 0.489 | -2.364 | 0.018* | -2.116 | -0.198 |
|  | datasetOriginal:settingdigilab | 0.488 | 0.625 | 0.781 | 0.435 | -0.737 | 1.712 |

**Supplementary Table S10.** Descriptive statistics of participant ratings of secondary appraisals, including aesthetic judgements, implications, and impact measures, by condition (overall & by experience type)

**S10A.** Summary statistics of aesthetic judgement ratings.

*Note.* All variables were rated on 7pt. bipolar scales. Higher values indicate agreement with the name of the variable (i.e., for ‘Liking’: 1 = ‘Something I disliked’, 7 = ‘Something I personally liked’).

| Condition | ExpType | *n* | Liking | | Beauty | | Goodness | | Meaning | | Complex | | Interest | | Strength | | Activity | |
| --- | --- | --- | --- | --- | --- | --- | --- | --- | --- | --- | --- | --- | --- | --- | --- | --- | --- | --- |
|  |  |  | *M* | *SD* | *M* | *SD* | *M* | *SD* | *M* | *SD* | *M* | *SD* | *M* | *SD* | *M* | *SD* | *M* | *SD* |
| Museum | Total | 745 | 4.84 | 1.79 | 5.07 | 1.57 | 5.34 | 1.57 | 4.90 | 1.66 | 4.92 | 1.70 | 5.30 | 1.64 | 5.06 | 1.58 | 4.55 | 1.78 |
| Digital | Total | 904 | 4.29 | 1.84 | 4.55 | 1.65 | 4.88 | 1.65 | 4.40 | 1.71 | 4.40 | 1.70 | 4.66 | 1.80 | 4.60 | 1.58 | 4.32 | 1.66 |
| Museum | Harmonious | 243 | 5.27 | 1.53 | 5.44 | 1.39 | 5.78 | 1.39 | 5.02 | 1.43 | 5.05 | 1.55 | 5.63 | 1.34 | 5.23 | 1.38 | 4.59 | 1.60 |
|  | Novel | 119 | 5.86 | 1.43 | 6.03 | 1.18 | 6.23 | 1.18 | 5.61 | 1.34 | 5.18 | 1.64 | 6.06 | 1.14 | 5.80 | 1.26 | 5.25 | 1.53 |
|  | Transformative | 53 | 5.16 | 1.71 | 4.98 | 1.60 | 5.55 | 1.60 | 6.02 | 1.28 | 5.74 | 1.33 | 6.08 | 1.36 | 5.94 | 1.42 | 5.31 | 1.84 |
|  | Disengaged | 230 | 3.98 | 1.78 | 4.38 | 1.55 | 4.53 | 1.55 | 3.98 | 1.74 | 4.25 | 1.81 | 4.32 | 1.76 | 4.15 | 1.55 | 3.93 | 1.82 |
|  | Negative | 100 | 4.34 | 1.85 | 4.62 | 1.60 | 4.94 | 1.60 | 5.30 | 1.57 | 5.38 | 1.54 | 5.40 | 1.65 | 5.35 | 1.60 | 4.63 | 1.86 |
| Digital | Harmonious | 253 | 4.97 | 1.26 | 5.14 | 1.25 | 5.27 | 1.25 | 4.62 | 1.30 | 4.40 | 1.53 | 5.16 | 1.27 | 4.81 | 1.22 | 4.43 | 1.41 |
|  | Novel | 183 | 6.02 | 1.04 | 5.83 | 1.19 | 5.97 | 1.19 | 5.42 | 1.48 | 4.94 | 1.69 | 5.80 | 1.42 | 5.63 | 1.28 | 5.16 | 1.49 |
|  | Transformative | 70 | 4.04 | 1.71 | 4.17 | 1.50 | 5.01 | 1.50 | 5.03 | 1.48 | 4.89 | 1.48 | 4.93 | 1.58 | 5.07 | 1.44 | 5.03 | 1.39 |
|  | Disengaged | 304 | 3.18 | 1.58 | 3.77 | 1.50 | 4.09 | 1.50 | 3.49 | 1.72 | 3.96 | 1.77 | 3.64 | 1.86 | 3.75 | 1.53 | 3.57 | 1.63 |
|  | Negative | 94 | 2.82 | 1.70 | 3.27 | 1.58 | 4.12 | 1.58 | 4.27 | 1.77 | 4.43 | 1.76 | 4.23 | 1.79 | 4.40 | 1.75 | 4.30 | 1.81 |

**S10B.** Summary statistics of implications and impact ratings.

*Note.* Item ‘Cognitive to Visceral’ was rated on a 7 pt. bipolar scale (1=’More detached and cognitive’, 7=’Very emotional and visceral’; item was reverse scored from original survey). Item ‘Pay to see again/in person’ was rated on a 7 pt. scale (1=‘Not at all’, 7=‘Very much so’). For the final columns (impact measures), items were rated on 11pt bipolar scales (0-10): Feel better (1=‘worse’ to 10='better’); Better person (1=‘worse person’ to 10=‘better person’); Change you (1=‘Not at all’ to 10=‘Very much so’).

| Condition | ExpType | *n* | Cognitive to Visceral | | Pay to see again / in person | | Feel better | | Better person | | Change you | |
| --- | --- | --- | --- | --- | --- | --- | --- | --- | --- | --- | --- | --- |
|  |  |  | *M* | *SD* | *M* | *SD* | *M* | *SD* | *M* | *SD* | *M* | *SD* |
| Museum | Total | 745 | 3.67 | 1.75 | 3.60 | 1.88 | 5.99 | 1.98 | 5.82 | 1.59 | 3.87 | 2.71 |
| Digital | Total | 904 | 3.75 | 1.72 | 3.24 | 1.99 | 5.52 | 2.08 | 5.31 | 1.23 | 3.18 | 2.54 |
| Museum | Harmonious | 243 | 3.75 | 1.51 | 3.85 | 1.69 | 6.48 | 1.75 | 5.87 | 1.41 | 3.93 | 2.49 |
|  | Novel | 119 | 4.52 | 1.74 | 4.97 | 1.57 | 7.59 | 1.87 | 6.85 | 1.92 | 5.39 | 2.72 |
|  | Transformative | 53 | 4.87 | 1.69 | 4.65 | 1.53 | 5.50 | 2.10 | 6.17 | 1.63 | 5.27 | 2.06 |
|  | Disengaged | 230 | 2.66 | 1.49 | 2.43 | 1.60 | 5.21 | 1.60 | 5.35 | 1.37 | 2.56 | 2.63 |
|  | Negative | 100 | 4.20 | 1.74 | 3.49 | 1.81 | 4.93 | 1.82 | 5.38 | 1.42 | 4.25 | 2.28 |
| Digital | Harmonious | 253 | 3.73 | 1.41 | 3.54 | 1.75 | 6.20 | 1.54 | 5.45 | 1.03 | 3.05 | 2.41 |
|  | Novel | 183 | 4.81 | 1.44 | 5.02 | 1.73 | 7.56 | 1.70 | 6.11 | 1.51 | 5.03 | 2.12 |
|  | Transformative | 70 | 4.67 | 1.62 | 3.79 | 1.86 | 4.54 | 2.07 | 5.24 | 1.26 | 4.76 | 1.88 |
|  | Disengaged | 304 | 2.84 | 1.56 | 2.05 | 1.45 | 4.60 | 1.34 | 4.93 | 0.74 | 1.87 | 2.24 |
|  | Negative | 94 | 3.95 | 1.96 | 2.44 | 1.77 | 3.38 | 1.86 | 4.65 | 1.45 | 3.03 | 2.22 |

**Supplementary Table S11.** Three-way ANOVA results considering judgement ratings across levels of condition, artwork, and experience type.

*Note.* Items ‘Liking’ to ‘Pay to see again/in person’ were rated on 7 pt. scales (1-7). Final items, ‘Feel better,’ ‘Better person,’ ‘Change you’ were rated on 11 pt. scales (0-10). Significance values were interpreted via Bonferroni correction (p = 0.05 / 13 analyses = 0.0038).

| Variable | Term | *df* | Sum Sq. | Mean Sq. | Statistic | *p* | η^2^ |
| --- | --- | --- | --- | --- | --- | --- | --- |
| Liking | Condition | 1 | 123.26 | 123.26 | 54.73 | < .001*** | 0.02 |
|  | Artwork | 7 | 249.21 | 35.60 | 15.81 | < .001*** | 0.04 |
|  | ExpType | 4 | 1,347.65 | 336.91 | 149.59 | < .001*** | 0.24 |
|  | Condition × Artwork | 7 | 86.84 | 12.41 | 5.51 | < .001*** | 0.02 |
|  | Condition × ExpType | 4 | 94.46 | 23.61 | 10.48 | < .001*** | 0.02 |
|  | Artwork × ExpType | 28 | 87.42 | 3.12 | 1.39 | .087 | 0.02 |
|  | Condition × Artwork × ExpType | 28 | 49.96 | 1.78 | 0.79 | .772 | 0.01 |
|  | Residuals | 1,556 | 3,504.49 | 2.25 |  |  | 0.63 |
| Beauty | Condition | 1 | 108.71 | 108.71 | 59.24 | < .001*** | 0.02 |
|  | Artwork | 7 | 380.80 | 54.40 | 29.64 | < .001*** | 0.09 |
|  | ExpType | 4 | 817.55 | 204.39 | 111.37 | < .001*** | 0.19 |
|  | Condition × Artwork | 7 | 52.01 | 7.43 | 4.05 | < .001*** | 0.01 |
|  | Condition × ExpType | 4 | 49.37 | 12.34 | 6.73 | < .001*** | 0.01 |
|  | Artwork × ExpType | 28 | 42.29 | 1.51 | 0.82 | .730 | 0.01 |
|  | Condition × Artwork × ExpType | 28 | 70.40 | 2.51 | 1.37 | .094 | 0.02 |
|  | Residuals | 1,566 | 2,873.88 | 1.84 |  |  | 0.65 |
| Goodness | Condition | 1 | 87.26 | 87.26 | 48.68 | < .001*** | 0.02 |
|  | Artwork | 7 | 264.76 | 37.82 | 21.10 | < .001*** | 0.07 |
|  | ExpType | 4 | 708.52 | 177.13 | 98.82 | < .001*** | 0.18 |
|  | Condition × Artwork | 7 | 70.40 | 10.06 | 5.61 | < .001*** | 0.02 |
|  | Condition × ExpType | 4 | 12.41 | 3.10 | 1.73 | .141 | 0.00 |
|  | Artwork × ExpType | 28 | 54.70 | 1.95 | 1.09 | .341 | 0.01 |
|  | Condition × Artwork × ExpType | 28 | 41.88 | 1.50 | 0.83 | .713 | 0.01 |
|  | Residuals | 1,564 | 2,803.31 | 1.79 |  |  | 0.69 |
| Meaning | Condition | 1 | 104.37 | 104.37 | 50.19 | < .001*** | 0.02 |
|  | Artwork | 7 | 478.69 | 68.38 | 32.88 | < .001*** | 0.10 |
|  | ExpType | 4 | 700.19 | 175.05 | 84.17 | < .001*** | 0.15 |
|  | Condition × Artwork | 7 | 121.29 | 17.33 | 8.33 | < .001*** | 0.03 |
|  | Condition × ExpType | 4 | 35.32 | 8.83 | 4.25 | .002** | 0.01 |
|  | Artwork × ExpType | 28 | 64.82 | 2.31 | 1.11 | .312 | 0.01 |
|  | Condition × Artwork × ExpType | 28 | 41.72 | 1.49 | 0.72 | .861 | 0.01 |
|  | Residuals | 1,566 | 3,256.81 | 2.08 |  |  | 0.68 |
| Complex | Condition | 1 | 109.50 | 109.50 | 46.61 | < .001*** | 0.02 |
|  | Artwork | 7 | 608.76 | 86.97 | 37.02 | < .001*** | 0.13 |
|  | ExpType | 4 | 204.71 | 51.18 | 21.79 | < .001*** | 0.04 |
|  | Condition × Artwork | 7 | 134.35 | 19.19 | 8.17 | < .001*** | 0.03 |
|  | Condition × ExpType | 4 | 31.34 | 7.83 | 3.33 | .010** | 0.01 |
|  | Artwork × ExpType | 28 | 62.74 | 2.24 | 0.95 | .534 | 0.01 |
|  | Condition × Artwork × ExpType | 28 | 41.16 | 1.47 | 0.63 | .937 | 0.01 |
|  | Residuals | 1,565 | 3,676.33 | 2.35 |  |  | 0.76 |
| Interest | Condition | 1 | 163.91 | 163.91 | 73.83 | < .001*** | 0.03 |
|  | Artwork | 7 | 311.67 | 44.52 | 20.05 | < .001*** | 0.06 |
|  | ExpType | 4 | 886.38 | 221.59 | 99.81 | < .001*** | 0.17 |
|  | Condition × Artwork | 7 | 99.05 | 14.15 | 6.37 | < .001*** | 0.02 |
|  | Condition × ExpType | 4 | 35.96 | 8.99 | 4.05 | .003** | 0.01 |
|  | Artwork × ExpType | 28 | 62.58 | 2.23 | 1.01 | .456 | 0.01 |
|  | Condition × Artwork × ExpType | 28 | 52.53 | 1.88 | 0.84 | .698 | 0.01 |
|  | Residuals | 1,567 | 3,478.95 | 2.22 |  |  | 0.68 |
| Strength | Condition | 1 | 86.78 | 86.78 | 46.23 | < .001*** | 0.02 |
|  | Artwork | 7 | 278.12 | 39.73 | 21.17 | < .001*** | 0.07 |
|  | ExpType | 4 | 680.63 | 170.16 | 90.64 | < .001*** | 0.16 |
|  | Condition × Artwork | 7 | 71.66 | 10.24 | 5.45 | < .001*** | 0.02 |
|  | Condition × ExpType | 4 | 24.30 | 6.07 | 3.24 | .012* | 0.01 |
|  | Artwork × ExpType | 28 | 67.44 | 2.41 | 1.28 | .147 | 0.02 |
|  | Condition × Artwork × ExpType | 28 | 47.51 | 1.70 | 0.90 | .611 | 0.01 |
|  | Residuals | 1,566 | 2,939.77 | 1.88 |  |  | 0.70 |
| Activity | Condition | 1 | 21.55 | 21.55 | 9.55 | .002** | 0.00 |
|  | Artwork | 7 | 624.96 | 89.28 | 39.58 | < .001*** | 0.13 |
|  | ExpType | 4 | 459.82 | 114.96 | 50.96 | < .001*** | 0.09 |
|  | Condition × Artwork | 7 | 106.30 | 15.19 | 6.73 | < .001*** | 0.02 |
|  | Condition × ExpType | 4 | 7.73 | 1.93 | 0.86 | .490 | 0.00 |
|  | Artwork × ExpType | 28 | 52.90 | 1.89 | 0.84 | .709 | 0.01 |
|  | Condition × Artwork × ExpType | 28 | 37.55 | 1.34 | 0.59 | .954 | 0.01 |
|  | Residuals | 1,566 | 3,532.74 | 2.26 |  |  | 0.73 |
| Cognitive to Visceral | Condition | 1 | 2.27 | 2.27 | 0.97 | .325 | 0.00 |
|  | Artwork | 7 | 228.78 | 32.68 | 13.92 | < .001*** | 0.05 |
|  | ExpType | 4 | 862.96 | 215.74 | 91.91 | < .001*** | 0.18 |
|  | Condition × Artwork | 7 | 20.63 | 2.95 | 1.26 | .269 | 0.00 |
|  | Condition × ExpType | 4 | 14.95 | 3.74 | 1.59 | .174 | 0.00 |
|  | Artwork × ExpType | 28 | 76.79 | 2.74 | 1.17 | .249 | 0.02 |
|  | Condition × Artwork × ExpType | 28 | 63.75 | 2.28 | 0.97 | .510 | 0.01 |
|  | Residuals | 1,551 | 3,640.72 | 2.35 |  |  | 0.74 |
| Pay to see again / in person | Condition | 1 | 50.27 | 50.27 | 19.13 | < .001*** | 0.01 |
|  | Artwork | 7 | 276.28 | 39.47 | 15.02 | < .001*** | 0.04 |
|  | ExpType | 4 | 1,524.08 | 381.02 | 145.02 | < .001*** | 0.24 |
|  | Condition × Artwork | 7 | 105.74 | 15.11 | 5.75 | < .001*** | 0.02 |
|  | Condition × ExpType | 4 | 46.16 | 11.54 | 4.39 | .002** | 0.01 |
|  | Artwork × ExpType | 28 | 64.26 | 2.29 | 0.87 | .657 | 0.01 |
|  | Condition × Artwork × ExpType | 28 | 68.37 | 2.44 | 0.93 | .572 | 0.01 |
|  | Residuals | 1,556 | 4,088.18 | 2.63 |  |  | 0.66 |
| Feel better | Condition | 1 | 90.49 | 90.49 | 32.96 | < .001*** | 0.01 |
|  | Artwork | 7 | 356.36 | 50.91 | 18.54 | < .001*** | 0.05 |
|  | ExpType | 4 | 1,852.20 | 463.05 | 168.65 | < .001*** | 0.27 |
|  | Condition × Artwork | 7 | 26.81 | 3.83 | 1.40 | .203 | 0.00 |
|  | Condition × ExpType | 4 | 68.14 | 17.03 | 6.20 | < .001*** | 0.01 |
|  | Artwork × ExpType | 28 | 76.65 | 2.74 | 1.00 | .470 | 0.01 |
|  | Condition × Artwork × ExpType | 28 | 125.52 | 4.48 | 1.63 | .020* | 0.02 |
|  | Residuals | 1,553 | 4,264.02 | 2.75 |  |  | 0.62 |
| Better person | Condition | 1 | 103.25 | 103.25 | 59.31 | < .001*** | 0.03 |
|  | Artwork | 7 | 33.73 | 4.82 | 2.77 | .007** | 0.01 |
|  | ExpType | 4 | 372.18 | 93.04 | 53.45 | < .001*** | 0.11 |
|  | Condition × Artwork | 7 | 16.34 | 2.33 | 1.34 | .227 | 0.00 |
|  | Condition × ExpType | 4 | 11.48 | 2.87 | 1.65 | .159 | 0.00 |
|  | Artwork × ExpType | 28 | 42.56 | 1.52 | 0.87 | .657 | 0.01 |
|  | Condition × Artwork × ExpType | 28 | 32.12 | 1.15 | 0.66 | .913 | 0.01 |
|  | Residuals | 1,551 | 2,699.84 | 1.74 |  |  | 0.82 |
| Change you | Condition | 1 | 189.74 | 189.74 | 33.78 | < .001*** | 0.02 |
|  | Artwork | 7 | 140.26 | 20.04 | 3.57 | .001*** | 0.01 |
|  | ExpType | 4 | 1,987.95 | 496.99 | 88.47 | < .001*** | 0.17 |
|  | Condition × Artwork | 7 | 20.34 | 2.91 | 0.52 | .822 | 0.00 |
|  | Condition × ExpType | 4 | 22.84 | 5.71 | 1.02 | .397 | 0.00 |
|  | Artwork × ExpType | 28 | 165.91 | 5.93 | 1.05 | .388 | 0.01 |
|  | Condition × Artwork × ExpType | 27 | 118.66 | 4.39 | 0.78 | .779 | 0.01 |
|  | Residuals | 1,553 | 8,724.15 | 5.62 |  |  | 0.77 |

**Supplementary Table S12.** Descriptive statistics of phenomenal state ratings, by condition (overall & by experience type).

*Note.* All items were rated on an 11 pt. scale (0=‘Not at all’, 10=‘Very much so’), in response to the prompt ‘While viewing the artwork, I felt…’

**S12A.** Ratings of first eight of sixteen phenomenal states.

| Condition | ExpType | *n* | Love | | Happiness | | Playfulness | | Pleasure | | Sense of beauty | | Insight | | Moved | | Transformation | |
| --- | --- | --- | --- | --- | --- | --- | --- | --- | --- | --- | --- | --- | --- | --- | --- | --- | --- | --- |
|  |  |  | *M* | *SD* | *M* | *SD* | *M* | *SD* | *M* | *SD* | *M* | *SD* | *M* | *SD* | *M* | *SD* | *M* | *SD* |
| Museum | Total | 745 | 2.86 | 3.11 | 3.70 | 3.11 | 2.92 | 1.57 | 4.55 | 3.09 | 5.09 | 3.22 | 3.16 | 2.96 | 3.88 | 3.01 | 2.57 | 2.79 |
| Digital | Total | 904 | 2.34 | 2.75 | 3.25 | 2.99 | 3.53 | 1.65 | 3.92 | 3.12 | 4.64 | 3.08 | 2.47 | 2.78 | 3.45 | 2.82 | 2.32 | 2.66 |
| Museum | Harmonious | 243 | 3.32 | 2.65 | 5.29 | 2.32 | 3.63 | 1.39 | 6.15 | 2.23 | 6.63 | 2.31 | 3.02 | 2.69 | 4.23 | 2.67 | 2.10 | 2.25 |
|  | Novel | 119 | 7.17 | 2.22 | 7.20 | 2.04 | 5.89 | 1.18 | 7.87 | 1.53 | 8.22 | 1.80 | 5.59 | 2.85 | 6.61 | 2.22 | 5.30 | 2.74 |
|  | Transformative | 53 | 4.13 | 3.01 | 3.06 | 3.03 | 3.06 | 1.60 | 4.57 | 2.92 | 5.94 | 2.50 | 6.19 | 1.92 | 6.62 | 2.24 | 6.02 | 1.80 |
|  | Disengaged | 230 | 0.54 | 1.12 | 1.36 | 1.83 | 1.07 | 1.55 | 2.37 | 2.07 | 2.55 | 2.44 | 1.31 | 1.87 | 1.72 | 2.04 | 0.88 | 1.74 |
|  | Negative | 100 | 1.29 | 1.83 | 1.40 | 1.60 | 1.87 | 1.60 | 1.70 | 1.94 | 3.00 | 2.57 | 3.25 | 2.91 | 3.29 | 2.81 | 2.48 | 2.46 |
| Digital | Harmonious | 253 | 2.44 | 2.16 | 4.39 | 2.15 | 4.25 | 1.25 | 5.26 | 2.02 | 5.94 | 2.10 | 2.12 | 2.28 | 3.55 | 2.18 | 1.78 | 1.95 |
|  | Novel | 183 | 5.75 | 2.60 | 7.14 | 1.95 | 6.42 | 1.19 | 7.79 | 1.69 | 7.88 | 1.76 | 4.65 | 2.97 | 6.36 | 2.19 | 5.02 | 2.73 |
|  | Transformative | 70 | 3.27 | 2.62 | 2.66 | 1.88 | 4.16 | 1.50 | 3.71 | 2.39 | 4.93 | 2.57 | 5.43 | 2.31 | 5.87 | 2.32 | 5.17 | 2.32 |
|  | Disengaged | 304 | 0.51 | 1.05 | 0.78 | 1.15 | 1.49 | 1.50 | 1.40 | 1.63 | 2.36 | 2.18 | 0.86 | 1.65 | 1.20 | 1.58 | 0.56 | 1.13 |
|  | Negative | 94 | 0.69 | 1.16 | 1.03 | 1.27 | 2.04 | 1.58 | 1.10 | 1.36 | 2.03 | 2.18 | 2.21 | 2.43 | 2.94 | 2.20 | 2.14 | 2.12 |

**S12A.** Ratings of second eight of sixteen phenomenal states.

| Condition | ExpType | *n* | Change in myself | | Thrilled | | Active engagement | | Boredom | | Shock | | Anger | | Sadness | | Anxiety | |
| --- | --- | --- | --- | --- | --- | --- | --- | --- | --- | --- | --- | --- | --- | --- | --- | --- | --- | --- |
|  |  |  | *M* | *SD* | *M* | *SD* | *M* | *SD* | *M* | *SD* | *M* | *SD* | *M* | *SD* | *M* | *SD* | *M* | *SD* |
| Museum | Total | 745 | 1.89 | 2.45 | 2.47 | 2.80 | 4.45 | 3.15 | 1.92 | 2.52 | 1.72 | 2.46 | 1.35 | 2.29 | 3.00 | 3.18 | 2.02 | 2.75 |
| Digital | Total | 904 | 2.24 | 2.54 | 3.08 | 2.77 | 4.88 | 3.10 | 2.06 | 2.61 | 1.42 | 2.28 | 1.04 | 1.91 | 2.10 | 2.60 | 1.61 | 2.39 |
| Museum | Harmonious | 243 | 1.23 | 1.65 | 2.38 | 2.42 | 4.78 | 2.87 | 1.21 | 1.88 | 1.06 | 1.70 | 0.42 | 1.06 | 2.03 | 2.70 | 1.02 | 1.72 |
|  | Novel | 119 | 4.81 | 2.73 | 5.46 | 2.97 | 7.03 | 2.56 | 1.22 | 1.71 | 2.54 | 2.65 | 1.08 | 1.62 | 3.15 | 2.88 | 1.47 | 1.86 |
|  | Transformative | 53 | 4.21 | 2.54 | 4.68 | 2.75 | 6.13 | 2.31 | 2.62 | 2.63 | 5.74 | 2.51 | 5.87 | 2.33 | 6.89 | 2.59 | 6.28 | 2.55 |
|  | Disengaged | 230 | 0.47 | 1.12 | 0.62 | 1.31 | 2.67 | 2.82 | 2.56 | 2.92 | 0.44 | 1.12 | 0.40 | 1.02 | 1.67 | 2.32 | 0.87 | 1.72 |
|  | Negative | 100 | 2.08 | 2.14 | 2.21 | 2.19 | 3.77 | 2.80 | 2.68 | 2.96 | 3.17 | 2.74 | 3.71 | 2.74 | 6.15 | 2.80 | 5.46 | 2.78 |
| Digital | Harmonious | 253 | 1.66 | 1.84 | 2.93 | 2.27 | 5.27 | 2.65 | 1.43 | 2.02 | 0.56 | 1.01 | 0.37 | 0.85 | 1.42 | 1.91 | 0.78 | 1.31 |
|  | Novel | 183 | 4.98 | 2.40 | 5.62 | 2.50 | 7.54 | 2.17 | 0.97 | 1.60 | 1.44 | 2.08 | 0.42 | 0.91 | 2.34 | 2.70 | 1.21 | 1.80 |
|  | Transformative | 70 | 5.00 | 2.23 | 5.79 | 2.22 | 6.50 | 2.35 | 2.33 | 2.17 | 5.43 | 2.44 | 4.70 | 2.46 | 5.43 | 2.73 | 5.97 | 2.36 |
|  | Disengaged | 304 | 0.55 | 1.14 | 1.03 | 1.46 | 2.88 | 2.61 | 2.95 | 3.15 | 0.54 | 1.41 | 0.46 | 1.08 | 1.04 | 1.72 | 0.53 | 1.02 |
|  | Negative | 94 | 1.88 | 1.86 | 3.21 | 2.44 | 3.89 | 2.86 | 2.81 | 2.76 | 3.55 | 2.71 | 3.19 | 2.41 | 4.36 | 2.81 | 4.84 | 2.49 |

**Supplementary Table S13.** Pairwise Contrasts across levels of artwork and experience type, following three-way ANOVA of overall magnitude of phenomenal state ratings.

*Note*. Full three-way ANOVA results reported in Table 4. Overall magnitude refers to ratings averaged across all 16 phenomenal states (each rated on 11 pt. scale, 0-10).

**S13A.** Pairwise contrasts between experience types, within levels of artwork (averaged across levels of condition).

| Artwork | ExpType Contrasts | estimate | SE | df | t.ratio | p.value |
| --- | --- | --- | --- | --- | --- | --- |
| Basquiat | Harmonious-Novel | -1.70095 | 0.203 | 26297 | -8.365 | <.0001 |
|  | Harmonious-Transformative | -1.75184 | 0.181 | 26297 | -9.692 | <.0001 |
|  | Harmonious-Disengaged | 1.65621 | 0.176 | 26297 | 9.413 | <.0001 |
|  | Harmonious-Negative | 0.32407 | 0.17 | 26297 | 1.91 | 0.3119 |
|  | Novel-Transformative | -0.05089 | 0.176 | 26297 | -0.288 | 0.9985 |
|  | Novel-Disengaged | 3.35717 | 0.171 | 26297 | 19.577 | <.0001 |
|  | Novel-Negative | 2.02502 | 0.165 | 26297 | 12.27 | <.0001 |
|  | Transformative-Disengaged | 3.40806 | 0.144 | 26297 | 23.671 | <.0001 |
|  | Transformative-Negative | 2.07591 | 0.136 | 26297 | 15.237 | <.0001 |
|  | Disengaged-Negative | -1.33215 | 0.13 | 26297 | -10.262 | <.0001 |
| Katz | Harmonious-Novel | -2.10628 | 0.148 | 26297 | -14.188 | <.0001 |
|  | Harmonious-Transformative | -2.28411 | 0.315 | 26297 | -7.248 | <.0001 |
|  | Harmonious-Disengaged | 1.56495 | 0.106 | 26297 | 14.786 | <.0001 |
|  | Harmonious-Negative | -0.44408 | 0.185 | 26297 | -2.399 | 0.1154 |
|  | Novel-Transformative | -0.17783 | 0.331 | 26297 | -0.538 | 0.9834 |
|  | Novel-Disengaged | 3.67123 | 0.145 | 26297 | 25.25 | <.0001 |
|  | Novel-Negative | 1.6622 | 0.21 | 26297 | 7.907 | <.0001 |
|  | Transformative-Disengaged | 3.84906 | 0.314 | 26297 | 12.269 | <.0001 |
|  | Transformative-Negative | 1.84003 | 0.349 | 26297 | 5.28 | <.0001 |
|  | Disengaged-Negative | -2.00903 | 0.183 | 26297 | -11.001 | <.0001 |
| Kupka | Harmonious-Novel | -1.63325 | 0.151 | 26297 | -10.822 | <.0001 |
|  | Harmonious-Transformative | -2.34387 | 0.226 | 26297 | -10.371 | <.0001 |
|  | Harmonious-Disengaged | 1.82387 | 0.115 | 26297 | 15.898 | <.0001 |
|  | Harmonious-Negative | 0.35144 | 0.187 | 26297 | 1.876 | 0.3302 |
|  | Novel-Transformative | -0.71062 | 0.244 | 26297 | -2.91 | 0.0297 |
|  | Novel-Disengaged | 3.45712 | 0.147 | 26297 | 23.455 | <.0001 |
|  | Novel-Negative | 1.98469 | 0.209 | 26297 | 9.5 | <.0001 |
|  | Transformative-Disengaged | 4.16775 | 0.224 | 26297 | 18.634 | <.0001 |
|  | Transformative-Negative | 2.69531 | 0.268 | 26297 | 10.048 | <.0001 |
|  | Disengaged-Negative | -1.47244 | 0.184 | 26297 | -7.981 | <.0001 |
| Magritte | Harmonious-Novel | -2.27753 | 0.132 | 26297 | -17.29 | <.0001 |
|  | Harmonious-Transformative | -2.27145 | 0.196 | 26297 | -11.604 | <.0001 |
|  | Harmonious-Disengaged | 1.56727 | 0.122 | 26297 | 12.829 | <.0001 |
|  | Harmonious-Negative | -0.03459 | 0.16 | 26297 | -0.217 | 0.9995 |
|  | Novel-Transformative | 0.00608 | 0.197 | 26297 | 0.031 | 1 |
|  | Novel-Disengaged | 3.8448 | 0.125 | 26297 | 30.778 | <.0001 |
|  | Novel-Negative | 2.24294 | 0.162 | 26297 | 13.861 | <.0001 |
|  | Transformative-Disengaged | 3.83872 | 0.191 | 26297 | 20.074 | <.0001 |
|  | Transformative-Negative | 2.23686 | 0.217 | 26297 | 10.302 | <.0001 |
|  | Disengaged-Negative | -1.60186 | 0.154 | 26297 | -10.392 | <.0001 |
| Miro | Harmonious-Novel | -2.04853 | 0.128 | 26297 | -16.049 | <.0001 |
|  | Harmonious-Transformative | -2.18452 | 0.223 | 26297 | -9.782 | <.0001 |
|  | Harmonious-Disengaged | 1.68607 | 0.115 | 26297 | 14.712 | <.0001 |
|  | Harmonious-Negative | 0.20777 | 0.189 | 26297 | 1.097 | 0.8083 |
|  | Novel-Transformative | -0.13599 | 0.233 | 26297 | -0.583 | 0.9776 |
|  | Novel-Disengaged | 3.7346 | 0.133 | 26297 | 28.139 | <.0001 |
|  | Novel-Negative | 2.25631 | 0.201 | 26297 | 11.23 | <.0001 |
|  | Transformative-Disengaged | 3.87059 | 0.226 | 26297 | 17.106 | <.0001 |
|  | Transformative-Negative | 2.3923 | 0.272 | 26297 | 8.797 | <.0001 |
|  | Disengaged-Negative | -1.47829 | 0.193 | 26297 | -7.664 | <.0001 |
| Monet | Harmonious-Novel | -1.77942 | 0.11 | 26297 | -16.122 | <.0001 |
|  | Harmonious-Transformative | -1.23006 | 0.478 | 26297 | -2.575 | 0.0749 |
|  | Harmonious-Disengaged | 1.35919 | 0.127 | 26297 | 10.694 | <.0001 |
|  | Harmonious-Negative | -0.58423 | 0.392 | 26297 | -1.491 | 0.5683 |
|  | Novel-Transformative | 0.54935 | 0.481 | 26297 | 1.141 | 0.7846 |
|  | Novel-Disengaged | 3.1386 | 0.141 | 26297 | 22.332 | <.0001 |
|  | Novel-Negative | 1.19519 | 0.396 | 26297 | 3.015 | 0.0216 |
|  | Transformative-Disengaged | 2.58925 | 0.485 | 26297 | 5.333 | <.0001 |
|  | Transformative-Negative | 0.64583 | 0.611 | 26297 | 1.057 | 0.8283 |
|  | Disengaged-Negative | -1.94342 | 0.401 | 26297 | -4.842 | <.0001 |
| Rothko | Harmonious-Novel | -2.11637 | 0.187 | 26297 | -11.336 | <.0001 |
|  | Harmonious-Transformative | -1.90543 | 0.228 | 26297 | -8.342 | <.0001 |
|  | Harmonious-Disengaged | 1.73407 | 0.122 | 26297 | 14.157 | <.0001 |
|  | Harmonious-Negative | 0.01318 | 0.162 | 26297 | 0.082 | 1 |
|  | Novel-Transformative | 0.21094 | 0.266 | 26297 | 0.794 | 0.9325 |
|  | Novel-Disengaged | 3.85044 | 0.183 | 26297 | 21.055 | <.0001 |
|  | Novel-Negative | 2.12955 | 0.211 | 26297 | 10.086 | <.0001 |
|  | Transformative-Disengaged | 3.6395 | 0.225 | 26297 | 16.153 | <.0001 |
|  | Transformative-Negative | 1.91861 | 0.249 | 26297 | 7.711 | <.0001 |
|  | Disengaged-Negative | -1.72089 | 0.157 | 26297 | -10.943 | <.0001 |
| Troger | Harmonious-Novel | -1.86017 | 0.165 | 26297 | -11.244 | <.0001 |
|  | Harmonious-Transformative | -1.83366 | 0.181 | 26297 | -10.153 | <.0001 |
|  | Harmonious-Disengaged | 1.66531 | 0.137 | 26297 | 12.139 | <.0001 |
|  | Harmonious-Negative | 0.55284 | 0.168 | 26297 | 3.3 | 0.0086 |
|  | Novel-Transformative | 0.02651 | 0.193 | 26297 | 0.137 | 0.9999 |
|  | Novel-Disengaged | 3.52547 | 0.154 | 26297 | 22.953 | <.0001 |
|  | Novel-Negative | 2.41301 | 0.181 | 26297 | 13.318 | <.0001 |
|  | Transformative-Disengaged | 3.49896 | 0.17 | 26297 | 20.604 | <.0001 |
|  | Transformative-Negative | 2.3865 | 0.195 | 26297 | 12.23 | <.0001 |
|  | Disengaged-Negative | -1.11246 | 0.156 | 26297 | -7.14 | <.0001 |

**S13B.** Pairwise contrasts between artworks, within levels of experience type (averaged across levels of condition).

| ExpType | Artwork Contrasts | estimate | SE | df | t.ratio | p.value |
| --- | --- | --- | --- | --- | --- | --- |
| Harmonious | Basquiat-Katz | 0.25364 | 0.166 | 26297 | 1.529 | 0.7917 |
|  | Basquiat-Kupka | 0.02695 | 0.169 | 26297 | 0.159 | 1 |
|  | Basquiat-Magritte | 0.10687 | 0.173 | 26297 | 0.619 | 0.9986 |
|  | Basquiat-Miro | 0.13099 | 0.165 | 26297 | 0.792 | 0.9936 |
|  | Basquiat-Monet | 0.25689 | 0.16 | 26297 | 1.601 | 0.7499 |
|  | Basquiat-Rothko | 0.14766 | 0.172 | 26297 | 0.857 | 0.9896 |
|  | Basquiat-Troger | -0.11125 | 0.181 | 26297 | -0.615 | 0.9987 |
|  | Katz-Kupka | -0.22669 | 0.115 | 26297 | -1.976 | 0.498 |
|  | Katz-Magritte | -0.14677 | 0.12 | 26297 | -1.224 | 0.9252 |
|  | Katz-Miro | -0.12265 | 0.109 | 26297 | -1.122 | 0.9523 |
|  | Katz-Monet | 0.00325 | 0.102 | 26297 | 0.032 | 1 |
|  | Katz-Rothko | -0.10598 | 0.119 | 26297 | -0.888 | 0.9872 |
|  | Katz-Troger | -0.36489 | 0.132 | 26297 | -2.77 | 0.1025 |
|  | Kupka-Magritte | 0.07992 | 0.124 | 26297 | 0.643 | 0.9983 |
|  | Kupka-Miro | 0.10404 | 0.114 | 26297 | 0.912 | 0.985 |
|  | Kupka-Monet | 0.22994 | 0.107 | 26297 | 2.154 | 0.3802 |
|  | Kupka-Rothko | 0.12071 | 0.124 | 26297 | 0.975 | 0.9779 |
|  | Kupka-Troger | -0.13819 | 0.136 | 26297 | -1.019 | 0.9718 |
|  | Magritte-Miro | 0.02412 | 0.119 | 26297 | 0.202 | 1 |
|  | Magritte-Monet | 0.15002 | 0.112 | 26297 | 1.335 | 0.8856 |
|  | Magritte-Rothko | 0.04079 | 0.129 | 26297 | 0.317 | 1 |
|  | Magritte-Troger | -0.21811 | 0.14 | 26297 | -1.557 | 0.7763 |
|  | Miro-Monet | 0.1259 | 0.101 | 26297 | 1.247 | 0.9179 |
|  | Miro-Rothko | 0.01667 | 0.119 | 26297 | 0.14 | 1 |
|  | Miro-Troger | -0.24224 | 0.131 | 26297 | -1.847 | 0.5879 |
|  | Monet-Rothko | -0.10923 | 0.112 | 26297 | -0.977 | 0.9777 |
|  | Monet-Troger | -0.36813 | 0.125 | 26297 | -2.948 | 0.0634 |
|  | Rothko-Troger | -0.2589 | 0.14 | 26297 | -1.854 | 0.583 |
| Novel | Basquiat-Katz | -0.15169 | 0.189 | 26297 | -0.801 | 0.9931 |
|  | Basquiat-Kupka | 0.09465 | 0.189 | 26297 | 0.502 | 0.9997 |
|  | Basquiat-Magritte | -0.4697 | 0.17 | 26297 | -2.762 | 0.1046 |
|  | Basquiat-Miro | -0.21659 | 0.174 | 26297 | -1.245 | 0.9185 |
|  | Basquiat-Monet | 0.17842 | 0.167 | 26297 | 1.07 | 0.9629 |
|  | Basquiat-Rothko | -0.26776 | 0.216 | 26297 | -1.241 | 0.9197 |
|  | Basquiat-Troger | -0.27046 | 0.19 | 26297 | -1.426 | 0.8454 |
|  | Katz-Kupka | 0.24634 | 0.178 | 26297 | 1.385 | 0.8646 |
|  | Katz-Magritte | -0.31802 | 0.158 | 26297 | -2.011 | 0.4742 |
|  | Katz-Miro | -0.0649 | 0.162 | 26297 | -0.4 | 0.9999 |
|  | Katz-Monet | 0.33011 | 0.155 | 26297 | 2.136 | 0.3914 |
|  | Katz-Rothko | -0.11607 | 0.206 | 26297 | -0.562 | 0.9993 |
|  | Katz-Troger | -0.11877 | 0.179 | 26297 | -0.663 | 0.9979 |
|  | Kupka-Magritte | -0.56436 | 0.157 | 26297 | -3.592 | 0.0079 |
|  | Kupka-Miro | -0.31124 | 0.161 | 26297 | -1.928 | 0.5313 |
|  | Kupka-Monet | 0.08377 | 0.153 | 26297 | 0.546 | 0.9994 |
|  | Kupka-Rothko | -0.36241 | 0.206 | 26297 | -1.762 | 0.6462 |
|  | Kupka-Troger | -0.36511 | 0.178 | 26297 | -2.049 | 0.4483 |
|  | Magritte-Miro | 0.25312 | 0.139 | 26297 | 1.817 | 0.6083 |
|  | Magritte-Monet | 0.64813 | 0.13 | 26297 | 4.985 | <.0001 |
|  | Magritte-Rothko | 0.20195 | 0.189 | 26297 | 1.069 | 0.9631 |
|  | Magritte-Troger | 0.19925 | 0.158 | 26297 | 1.258 | 0.9141 |
|  | Miro-Monet | 0.39501 | 0.135 | 26297 | 2.922 | 0.0683 |
|  | Miro-Rothko | -0.05117 | 0.192 | 26297 | -0.266 | 1 |
|  | Miro-Troger | -0.05387 | 0.163 | 26297 | -0.331 | 1 |
|  | Monet-Rothko | -0.44618 | 0.186 | 26297 | -2.401 | 0.2407 |
|  | Monet-Troger | -0.44888 | 0.155 | 26297 | -2.9 | 0.0726 |
|  | Rothko-Troger | -0.0027 | 0.207 | 26297 | -0.013 | 1 |
| Transformative | Basquiat-Katz | -0.27863 | 0.323 | 26297 | -0.862 | 0.9893 |
|  | Basquiat-Kupka | -0.56508 | 0.235 | 26297 | -2.405 | 0.2385 |
|  | Basquiat-Magritte | -0.41274 | 0.203 | 26297 | -2.033 | 0.459 |
|  | Basquiat-Miro | -0.30169 | 0.235 | 26297 | -1.284 | 0.905 |
|  | Basquiat-Monet | 0.77867 | 0.485 | 26297 | 1.606 | 0.7469 |
|  | Basquiat-Rothko | -0.00593 | 0.235 | 26297 | -0.025 | 1 |
|  | Basquiat-Troger | -0.19306 | 0.18 | 26297 | -1.07 | 0.9629 |
|  | Katz-Kupka | -0.28646 | 0.37 | 26297 | -0.773 | 0.9945 |
|  | Katz-Magritte | -0.13411 | 0.351 | 26297 | -0.382 | 0.9999 |
|  | Katz-Miro | -0.02307 | 0.37 | 26297 | -0.062 | 1 |
|  | Katz-Monet | 1.05729 | 0.563 | 26297 | 1.878 | 0.5665 |
|  | Katz-Rothko | 0.27269 | 0.37 | 26297 | 0.736 | 0.9959 |
|  | Katz-Troger | 0.08557 | 0.339 | 26297 | 0.253 | 1 |
|  | Kupka-Magritte | 0.15234 | 0.272 | 26297 | 0.56 | 0.9993 |
|  | Kupka-Miro | 0.26339 | 0.297 | 26297 | 0.888 | 0.9872 |
|  | Kupka-Monet | 1.34375 | 0.517 | 26297 | 2.597 | 0.157 |
|  | Kupka-Rothko | 0.55915 | 0.297 | 26297 | 1.886 | 0.5609 |
|  | Kupka-Troger | 0.37202 | 0.256 | 26297 | 1.456 | 0.8307 |
|  | Magritte-Miro | 0.11105 | 0.272 | 26297 | 0.408 | 0.9999 |
|  | Magritte-Monet | 1.19141 | 0.504 | 26297 | 2.365 | 0.2588 |
|  | Magritte-Rothko | 0.40681 | 0.272 | 26297 | 1.496 | 0.81 |
|  | Magritte-Troger | 0.21968 | 0.226 | 26297 | 0.97 | 0.9786 |
|  | Miro-Monet | 1.08036 | 0.517 | 26297 | 2.088 | 0.4229 |
|  | Miro-Rothko | 0.29576 | 0.297 | 26297 | 0.997 | 0.975 |
|  | Miro-Troger | 0.10863 | 0.256 | 26297 | 0.425 | 0.9999 |
|  | Monet-Rothko | -0.7846 | 0.517 | 26297 | -1.516 | 0.799 |
|  | Monet-Troger | -0.97173 | 0.495 | 26297 | -1.963 | 0.5075 |
|  | Rothko-Troger | -0.18713 | 0.256 | 26297 | -0.732 | 0.996 |
| Disengaged | Basquiat-Katz | 0.16237 | 0.121 | 26297 | 1.341 | 0.8832 |
|  | Basquiat-Kupka | 0.19461 | 0.125 | 26297 | 1.56 | 0.7744 |
|  | Basquiat-Magritte | 0.01792 | 0.127 | 26297 | 0.141 | 1 |
|  | Basquiat-Miro | 0.16084 | 0.129 | 26297 | 1.243 | 0.919 |
|  | Basquiat-Monet | -0.04014 | 0.146 | 26297 | -0.275 | 1 |
|  | Basquiat-Rothko | 0.22551 | 0.128 | 26297 | 1.766 | 0.6433 |
|  | Basquiat-Troger | -0.10216 | 0.13 | 26297 | -0.783 | 0.994 |
|  | Katz-Kupka | 0.03223 | 0.106 | 26297 | 0.304 | 1 |
|  | Katz-Magritte | -0.14445 | 0.108 | 26297 | -1.333 | 0.8864 |
|  | Katz-Miro | -0.00153 | 0.111 | 26297 | -0.014 | 1 |
|  | Katz-Monet | -0.20252 | 0.13 | 26297 | -1.553 | 0.7785 |
|  | Katz-Rothko | 0.06313 | 0.109 | 26297 | 0.578 | 0.9991 |
|  | Katz-Troger | -0.26453 | 0.113 | 26297 | -2.35 | 0.2665 |
|  | Kupka-Magritte | -0.17669 | 0.112 | 26297 | -1.571 | 0.7677 |
|  | Kupka-Miro | -0.03376 | 0.115 | 26297 | -0.293 | 1 |
|  | Kupka-Monet | -0.23475 | 0.134 | 26297 | -1.754 | 0.6516 |
|  | Kupka-Rothko | 0.0309 | 0.113 | 26297 | 0.273 | 1 |
|  | Kupka-Troger | -0.29676 | 0.117 | 26297 | -2.547 | 0.1759 |
|  | Magritte-Miro | 0.14292 | 0.118 | 26297 | 1.216 | 0.9275 |
|  | Magritte-Monet | -0.05806 | 0.136 | 26297 | -0.427 | 0.9999 |
|  | Magritte-Rothko | 0.20759 | 0.116 | 26297 | 1.794 | 0.624 |
|  | Magritte-Troger | -0.12008 | 0.119 | 26297 | -1.011 | 0.973 |
|  | Miro-Monet | -0.20099 | 0.138 | 26297 | -1.455 | 0.8314 |
|  | Miro-Rothko | 0.06467 | 0.118 | 26297 | 0.546 | 0.9994 |
|  | Miro-Troger | -0.263 | 0.121 | 26297 | -2.166 | 0.3728 |
|  | Monet-Rothko | 0.26565 | 0.137 | 26297 | 1.945 | 0.5197 |
|  | Monet-Troger | -0.06201 | 0.139 | 26297 | -0.445 | 0.9998 |
|  | Rothko-Troger | -0.32766 | 0.12 | 26297 | -2.739 | 0.1112 |
| Negative | Basquiat-Katz | -0.51451 | 0.189 | 26297 | -2.729 | 0.1139 |
|  | Basquiat-Kupka | 0.05432 | 0.188 | 26297 | 0.289 | 1 |
|  | Basquiat-Magritte | -0.25179 | 0.157 | 26297 | -1.608 | 0.7455 |
|  | Basquiat-Miro | 0.0147 | 0.193 | 26297 | 0.076 | 1 |
|  | Basquiat-Monet | -0.65141 | 0.396 | 26297 | -1.646 | 0.7219 |
|  | Basquiat-Rothko | -0.16323 | 0.159 | 26297 | -1.027 | 0.9705 |
|  | Basquiat-Troger | 0.11753 | 0.155 | 26297 | 0.757 | 0.9951 |
|  | Katz-Kupka | 0.56882 | 0.237 | 26297 | 2.4 | 0.2412 |
|  | Katz-Magritte | 0.26272 | 0.213 | 26297 | 1.233 | 0.9221 |
|  | Katz-Miro | 0.5292 | 0.241 | 26297 | 2.194 | 0.3552 |
|  | Katz-Monet | -0.1369 | 0.421 | 26297 | -0.325 | 1 |
|  | Katz-Rothko | 0.35128 | 0.215 | 26297 | 1.635 | 0.7288 |
|  | Katz-Troger | 0.63203 | 0.212 | 26297 | 2.981 | 0.0578 |
|  | Kupka-Magritte | -0.30611 | 0.212 | 26297 | -1.441 | 0.8384 |
|  | Kupka-Miro | -0.03962 | 0.241 | 26297 | -0.165 | 1 |
|  | Kupka-Monet | -0.70573 | 0.421 | 26297 | -1.676 | 0.7027 |
|  | Kupka-Rothko | -0.21755 | 0.214 | 26297 | -1.015 | 0.9723 |
|  | Kupka-Troger | 0.06321 | 0.212 | 26297 | 0.299 | 1 |
|  | Magritte-Miro | 0.26649 | 0.217 | 26297 | 1.227 | 0.924 |
|  | Magritte-Monet | -0.39962 | 0.408 | 26297 | -0.98 | 0.9774 |
|  | Magritte-Rothko | 0.08856 | 0.187 | 26297 | 0.473 | 0.9998 |
|  | Magritte-Troger | 0.36932 | 0.184 | 26297 | 2.005 | 0.4783 |
|  | Miro-Monet | -0.66611 | 0.423 | 26297 | -1.574 | 0.7664 |
|  | Miro-Rothko | -0.17793 | 0.219 | 26297 | -0.813 | 0.9925 |
|  | Miro-Troger | 0.10283 | 0.216 | 26297 | 0.476 | 0.9998 |
|  | Monet-Rothko | 0.48818 | 0.409 | 26297 | 1.194 | 0.9339 |
|  | Monet-Troger | 0.76894 | 0.407 | 26297 | 1.887 | 0.5596 |
|  | Rothko-Troger | 0.28076 | 0.186 | 26297 | 1.507 | 0.8038 |
